# Sucrose 6^F^-phosphate phosphorylase: a novel insight in the human gut microbiome

**DOI:** 10.1099/mgen.0.000253

**Published:** 2019-03-26

**Authors:** Alexandra S. Tauzin, Laetitia Bruel, Elisabeth Laville, Cendrine Nicoletti, David Navarro, Bernard Henrissat, Josette Perrier, Gabrielle Potocki-Veronese, Thierry Giardina, Mickael Lafond

**Affiliations:** ^1^​ Aix Marseille Univ, CNRS, Centrale Marseille, iSm2, Marseille, France; ^2^​ LISBP, CNRS, INRA, INSAT, Université de Toulouse, F-31400 Toulouse, France; ^3^​ INRA, Aix-Marseille Université, UMR1163, Biodiversité et Biotechnologie Fongiques, PolyTech, F-13009, Marseille, France; ^4^​ Architecture et Fonction des Macromolécules Biologiques, CNRS, Aix-Marseille Université, F-13288 Marseille, France; ^5^​ Department of Biological Sciences, King Abdulaziz University, 23218 Jeddah, Saudi Arabia; ^‡^​ Present address: LISBP, CNRS, INRA, INSAT, Université de Toulouse, F-31400 Toulouse, France.

**Keywords:** sucrose metabolism, sucrose phosphorylase, *Ruminococcus gnavus* E1, GH13_18, human gut microbiome

## Abstract

The human gut microbiome plays an essential role in maintaining human health including in degradation of dietary fibres and carbohydrates further used as nutrients by both the host and the gut bacteria. Previously, we identified a polysaccharide utilization loci (PUL) involved in sucrose and raffinose family oligosaccharide (RFO) metabolism from one of the most common *Firmicutes* present in individuals, *Ruminococcus gnavus* E1. One of the enzymes encoded by this PUL was annotated as a putative sucrose phosphate phosphorylase (*Rg*SPP). In the present study, we have in-depth characterized the heterologously expressed *Rg*SPP as sucrose 6^F^-phosphate phosphorylase (SPP), expanding our knowledge of the glycoside hydrolase GH13_18 subfamily. Specifically, the enzymatic characterization showed a selective activity on sucrose 6^F^-phosphate (S6^F^P) acting both in phosphorolysis releasing alpha-d-glucose-1-phosphate (G1P) and alpha-d-fructose-6-phosphate (F6P), and in reverse phosphorolysis from G1P and F6P to S6^F^P. Interestingly, such a SPP activity had never been observed in gut bacteria before. In addition, a phylogenetic and synteny analysis showed a clustering and a strictly conserved PUL organization specific to gut bacteria. However, a wide prevalence and abundance study with a human metagenomic library showed a correlation between SPP activity and the geographical origin of the individuals and, thus, most likely linked to diet. *Rgspp* gene overexpression has been observed in mice fed with a high-fat diet suggesting, as observed for humans, that intestine lipid and carbohydrate microbial metabolisms are intertwined. Finally, based on the genomic environment analysis, *in vitro* and *in vivo* studies, results provide new insights into the gut microbiota catabolism of sucrose, RFOs and S6^F^P.

## Data Summary

1. The *Ruminococcus gnavus* E1 sucrose 6^F^-phosphate phosphorylase (SPP) sequence is available from GenBank under accession number FQ790378 (see Fig. S5, available in the online version of this article).

2. All sequences, from the glycoside hydrolase GH13_18 subfamily used in the present study have been extracted from the CAZy database, URL: http://www.cazy.org.

3. Data used for the prevalence and abundance study were provided by the MetaHIT project (http://www.metahit.eu).

**Impact Statement**

The human gut microbiome is considered an organ due to its key and specific functions in host metabolism, host protection and immune-system development. It is also able to modulate its own species composition to aid resiliency and to re-establish an intestinal stability. Developments in (meta)genome sequencing technologies and bioinformatic tools have now enabled scientists to study the microbiome's complex composition, its specific functions and the bacteria–host interactions. Recently, polysaccharide utilization loci (PUL) from *Bacteroidetes* have been investigated, highlighting their ability to use complex polysaccharide as a carbon source. By contrast, studies about gene clusters from *Firmicutes* are fewer. This paper will be of interest to those working in the ﬁelds of bacterial genomics and metabolism, human gut microbiology and CAZymes. For the first time, to the best of our knowledge, the ability of a strict anaerobe *Firmicutes* strain (i.e. *Ruminococcus gnavus* E1), found in more than 90 % of healthy humans, to metabolize the sucrose 6^F^-phosphate originating from plants via a glycoside hydrolase GH13_18 enzyme located in a PUL has been proposed, paving the way to interesting new pathways in sucrose, raffinose family oligosaccharides and sucrose 6^F^-phosphate catabolisms. More broadly, the *Rgspp* gene abundance and expression has been, respectively, studied in a human metagenomic library and *in vivo* with a high-fat diet, to figure out the link between lipid and carbohydrate metabolisms.

## 

The human intestine is colonized by a complex, diverse and dynamic community of microorganisms, the so-called microbiome, which is in permanent interaction with the host [1]. It is now well established that the gut microbiome is of considerable interest for health. It is indeed involved in food degradation and assimilation, formation of bile salts, protection against pathogens, integrity of epithelial layer and immunity (for review see Sekirov *et al.*, 2010 [1]), while a gut microbiome imbalance (i.e. dysbiosis) has been associated with several metabolic diseases, like obesity [2]. Among the many functions attributed to the intestinal microbiota, the metabolism of carbohydrates is of crucial importance.

Sucrose [α-d-glucopyranosyl-(1,2)-β-d-fructofuranoside], which is one of the most abundant soluble carbohydrates in plant tissues and in processed food, is usually degraded into glucose and fructose by a sucrase isomaltase, an intestinal membrane linked enzyme produced by the host, and leads by further processing to the generation of energy in the form of ATP (for a review see Gericke *et al*. [3]). Sucrose is also a structural component of sucrosyl-oligosaccharides, including fructans (FOS, e.g. 1-kestose, nystose and fructo-furanosyl nystose) and the raffinose family oligosaccharides (RFOs, e.g. raffinose, stachyose and verbascose), which are both important classes of carbohydrates in the plant kingdom [4]. Conversely to sucrose, FOS and RFOs are resistant to the action of human enzymes in the digestive tract, but they can be cleaved by microbial gut enzymes inducing a beneficial effect for the host's health [5]. For example, bacterial α-galactosidases hydrolyse RFOs to release d-galactose and sucrose, which can also be metabolized by the resident microbiota. This microbial degradation relies on intracellular sucrose phosphorylases (SPs; EC 2.4.1.7).

Based on the CAZy classification (www.cazy.org [6]), the glycoside hydrolase 13 family members act on substrates containing α-glucoside linkages and consist of about 30 different enzyme specificities including glycoside hydrolases, transferases, isomerases and phosphorylases, subdivided in 42 subfamilies. All members share a conserved structural scaffold with seven highly conserved regions despite their low overall sequence identity [7, 8]. SPs belong to the glycoside hydrolase GH family 13 subfamily 18 (GH13_18) [9]. The GH13_18 members are retaining enzymes, which reversibly catalyse the reaction between sucrose and inorganic phosphate to synthesize alpha-d-glucose-1-phosphate (G1P) and d-fructose. The products are then slotted into microbial glycolytic pathways such as glycolysis. Due to their ability to perform *in vitro* reverse phosphorolysis from G1P and a wide range of acceptor molecules [10], SPs are used for biotechnological purposes, to produce, for example, a moisturizing agent (Glycoin [11]), glucosylated flavonoids (including resveratrol and quercetin [12, 13]) and rare disaccharides (including kojibiose and nigerose [14, 15]).

To date, only a few GH13_18 members have been biochemically characterized [16–27]. In 2004, the first 3D structure of the SP from *Bifidobacterium adolescentis* was solved [20]. In the last few years, new specificities have been described for members of the GH13_18 family. In 2014, Verhaeghe *et al*. revealed for the first time a GH13_18 from *Thermoanaerobacterium thermosaccharolyticum* allowing phosphorolysis of the 6^F^ phosphorylated sucrose (sucrose-6^F^-phosphate; S6^F^P), releasing G1P and alpha-d-fructose-6-phosphate (F6P) [25]. This enzyme was, thus, considered as a sucrose 6^F^-phosphate phosphorylase (SPP), which was the first and the only enzyme with this specificity reported so far. Furthermore, three members of the GH13_18 family were recently described as strict glucosylglycerate phosphorylases (GGaPs) [26]. The GGaPs from *Meiothermus silvanus*, *Spirochaeta thermophila* and *Escherichia coli* (i.e. *Ms*GGaPs, *St*GGaPs and *Ec*GGaPs, respectively) catalyse the reversible phosphorolysis of glucosylglycerate into G1P and d-glycerate. Very recently, an enzyme from *Marinobacter adhaerens* HP15 able to catalyse the reversible phosphorolysis of 2-*O*-α-d-glucosylglycerol has been characterized and named as glucosylglycerol phosphorylase (*Ma*GGoP [27]).

*Ruminococcus gnavus* E1 is a Gram-positive anaerobic bacterium belonging to the phylum Firmicutes. As one of the 57 most common species, it is present in around 90 % of individuals [28]. First isolated from the faeces of a healthy human [29], *Ruminococcus gnavus* E1 fosters a high interest due to its abilities: (i) to produce antimicrobial peptides active against *Clostridium perfringens* [30–32], (ii) to produce a large panel of glycoside hydrolases acting on dietary [33–35] and (iii) host mucin-glycans [36]. Two loci putatively involved in sucrose metabolism have previously been identified (i.e. *Rgaga1* and *Rgaga2*). Specifically, the *Rgaga2* locus, including genes putatively encoding a regulator, a GH13_31, two phosphotransferase system (PTS) sequences and a GH32, has been proposed to be involved in extracellular and intracellular sucrose assimilation [35]. Similarly, *Rgaga1* consists of six genes predicted to encode a transcriptional regulator (*agaR*), an ABC transporter (*agaE, H* and *G*), a SP (*sucP*) and a bifunctional α-galactosidase/sucrose kinase (*RgAgaSK*). *Rg*AgaSK is the only biochemically characterized bi-modular enzyme encoded by this locus so far. It produces sucrose 6-phosphate (S6P) or releases corresponding monosaccharides from sucrose or from RFOs based on sucrose kinase and α-galactosidase activities [33].

Here, we used an integrative approach to better understand the function and the metabolic role of the SucP enzyme (hereafter renamed as *Rg*SPP). Genomic environment analysis, in-depth biochemical characterization, analysis of gene prevalence in the human gut microbiome and *in vivo* studies highlighted the role of this enzyme in carbohydrate catabolism by the prominent gut bacterium *Ruminococcus gnavus* E1.

## Methods

### Materials

Oligonucleotides, Zeocin, Geneticin, ampicillin, kanamycin and all restriction DNA modifying enzymes (except DNA polymerase) were purchased from Invitrogen. Culture media were from BD Difco. PrimeSTAR HS DNA polymerase for PCRs was from Takara. *E. coli* DH5α (*sup*E44, *hsd*R17, *rec*A1, *end*A1, *gyr*A96, *thi*-1, *rel*A1) was used for the DNA procedures (Invitrogen). Sodium phosphate and potassium phosphate were purchased from Sigma–Aldrich, sodium acetate was from Calbiochem and sodium hydroxide was from Fischer Scientific. Ultracel system and Ultracel PES membrane were from Millipore. The protein assay reagent was from Bio-Rad. Standard diet (SD) and high-fat (HF) diet were provided by SAFE Laboratories.

### Cloning, expression and purification of *Rg*SPP

The *Rgspp* gene (accession no. FQ790378) was cloned from the genomic DNA of *Ruminococcus gnavus* E1 and amplified as described by Bruel *et al.* in 2011 with PCR reaction primers (Table S1) [33]. Recombinant *Rgspp*, with a C-terminal (His)_6_-tag, was synthesized from *E. coli* BL21(DE3) grown in LB broth containing 50 mg ampicillin l^−1^ as an overnight culture at 20 °C. Bacterial lysis was carried out with Cell Disruptor (Constant System) in the binding buffer for affinity chromatography (20 mM MOPS buffer pH 7.4, 40 mM imidazole) at 1.37 kbar. After centrifugation at 1500 ***g***, 30 min at 4 °C, the soluble fraction was loaded onto a Ni-NTA column and the recombinant protein was eluted with 125 mM imidazole in MOPS buffer pH 7.4. Fractions containing proteins were pooled, dialysed against HEPES buffer (50 mM, pH 7.0) and concentrated to 5 mg ml^−1^. The concentrated proteins were injected onto a Sephacryl S200 (26/60) size exclusion column (SEC) with flow rate of 1 ml min^−1^ and purified to near homogeneity (>90 %). The purity of the protein was checked by SDS-PAGE (12 %) [37], with an overall yield of 50 mg (l of culture)^−1^. The protein concentration was determined using the Bio-Rad protein assay kit with BSA as the standard.

### Enzyme assays

#### Phosphorolytic activity and kinetic parameters

Phosphorylase activity was measured using two different methods. In the first one, G1P concentration was indirectly quantified with 0.90 µM enzyme, by following, at 340 nm, the coupled production of NADPH from NADP^+^ and G1P by phosphoglucomutase and glucose-6-phosphate dehydrogenase [17]. In the second method, substrate and product concentrations were determined by High-Performance Anion-Exchange Chromatography coupled with Pulsed-Amperometric Detection (HPAEC-PAD). The phosphorylase activity assays were performed over 18 h using 0.18 µM enzyme. The protocol and gradient used are the same as described by Lafond *et al.* in 2011, except that buffer A was composed of 5 mM NaOAc and 80 mM NaOH [38]. Prior to being analysed, samples were inactivated by heating for 5 min at 70 °C. In both cases, reactions were realized with 20 mM sucrose or S6^F^P in 100 mM phosphate buffer pH 6.0 at 40 °C. The samples were injected on a Dionex system equipped with a GP40 gradient pump, an ED40 pulsed amperometric detector, an AS3500 auto-sampler (Thermo-Electron) and a CarboPac PA-100 analytical column at 25 °C (250×4 mm).

#### Specific activity

The specificity toward different substrates was analysed using 10 mM G1P as donor and 10 mM acceptor, i.e. d-fructose, d-glucose, d-galactose, d-xylose, d-leucrose, d-isomaltulose, d-fructose 6-phosphate, d-glucose 6-phosphate, d-galactose 6-phosphate, d-glucose 1,6-diphosphate, d-fructose 6-diphosphate, and 100 mM maltotetraose and maltoheptaose. All reactions were monitored in 50 mM MOPS buffer pH 6.0 at 40 °C, for 18 h, and with 0.18 or 0.64 µM enzyme.

#### Kinetic parameters

Kinetic parameters were determined using the same conditions described previously with production of NADPH and S6^F^P as the substrate in 100 mM sodium phosphate buffer, pH 6.0, at 40 °C and by incubating 0.1 µM enzyme with seven different concentrations of S6^F^P (1.0 to 20 mM) in a reaction volume of 500 µl. *K*_m_ and *k_cat_* values were calculated using the nonlinear regression Michaelis–Menten equation.

#### Effect of temperature and pH on enzyme activity

Optimal pH for *Rg*SPP was determined using the coupled method with S6^F^P in 50 mM sodium phosphate buffer in a pH range of 5.0 to 8.0 at 45 °C and with 0.92 µM enzyme. Optimal temperatures were also determined using the same enzymatic assay in 50 mM sodium phosphate buffer pH 6.0 and temperatures ranging from 20 to 80 °C.

### Caecal content (CC) collection and extraction of RNA

The *in vivo* assays included an experimental model of infection (C57BL6 gnotobiotic mice) subcontracted to Germ Free Animals Facility ANAXEM platform INRA, UMR 1319 Micalis, France. Animal experiments were performed according to the guidelines of the French Ethics Committee, i.e. agreement number A78-322-6 for mice maintained for 10 days on a control diet and agreement number A78-718 for mice maintained for 2 months on a HF diet (Table S2). C57BL6 mouse strains are established models for diet-induced obesity [39]. Mice were reared in Trexler type isolators (LaCalhène), fed *ad libitum* with commercial diets sterilized by gamma ray and supplied with sterile autoclaved drinking water. Male mice (8–9 weeks old) sourced by ANEXEM and maintained on SD were inoculated with *Ruminococcus gnavus* E1 (0.5 ml late log-phase culture at 10^8^ cells ml^−1^), by intra-gastric route, on 3 consecutive days in order to generate *Ruminococcus gnavus* E1 monoxenic mice. Then mice were randomly divided into two separate groups. The first group of *Ruminococcus gnavus* E1 monoxenic mice (*n*=10) was assigned for 10 days on a SD to obtain an effective colonization of the digestive tract by *Ruminococcus gnavus* E1, according to Graziani *et al*. [40]. The second group (*n*=10) was assigned for 2 months on a HF diet containing 34.9/100 g fat, 26/100 g carbohydrate and 26/100 g protein provided by SAFE Laboratories (Table S2). The C57BL6 mouse strain has proved particularly useful as the mice readily gain weight when fed HF diets, and are more susceptible to obesity and glucose intolerance when fed HF diets for at least 2 months [41–43]. After 10 days (i.e. for the SD) or 2 months (i.e. for the HF diet), individual faecal samples were collected and bacterial counts estimated. Colonization by *Ruminococcus gnavus* E1 was controlled and analysed by plating serial dilutions of suspensions obtained from ground colon in an anaerobic chamber using brain-heart infusion media, whereas the analyses of bacterial c.f.u. were performed with the CC samples. Body weight was also monitored every 2 weeks and food consumption was monitored weekly. Then, the animals were sacrificed, and faeces and the CCs were collected in 1 ml Tri-Reagent (Molecular Research Center) and frozen at −80 °C until RNA extraction was carried out.

### DNA isolation and PCR analysis of *Ruminococcus* gnavus E1

For each analysis, chromosomal DNA was isolated from 100 to 120 mg mouse faeces, using the Fast DNA SPIN kit for faeces (MP). Amplification of 16S rDNA was performed using oligonucleotide primers 16F8 and 16R1541, which corresponded to bacterial 16S rRNA gene conserved sequences (from positions 8 to 1541 on the *E. coli* 16S rRNA). The PCR conditions used were: DNA (50 ng); annealing at 60 °C (20 s), polymerization at 72 °C (30 s) and denaturation at 94 °C (20 s). Amplification reactions (30 cycles) were carried out in a Mastercycler Nexus GX2 (Eppendorf). Resulting DNA fragments were then sequenced.

### RNA isolation and quantitative Reverse Transcription-PCR analysis of the faeces and the CCs

Two hundred milligrams fresh material (faeces/CCs) were used for total RNA extraction according to the protocol described by Doré *et al*. and cleaned up with the RNeasy mini kit (Qiagen) [44]. RNA was spectrophotometrically quantified (260 nm) and purity assessed by the *A*_260_/*A*_280_ ratio using a NanoDrop 2000c UV-Vis spectrophotometer (Thermo Fischer Scientific). Reverse transcription of 6.25 ng RNA was performed with qScript cDNA (Quantabio) and 50 ng random primers in a 20 µl volume at 42 °C for 1 h with reverse transcriptase. All procedures were according to the manufacturer’s instructions. PCR was performed in the presence of SYBR green reagent with a Light Cycler 480 (Roche Technology) and carried out with 25 ng cDNA in 20 µl containing 10 µl PCR master mix reagent and 0.5 µM each primer. The sequences of the primers corresponding to the genes in mice were designed using the Universal Probe Library assay design centre (Roche) and are reported in Table S1. Thermal cycling conditions were as follows: 5 min denaturation at 94 °C; followed by 45 cycles of 10 s at 94 °C, 10 s at 60 °C and 10 s at 72 °C. Data were collected using the Light Cycler 480 software (Platform AVB, iSm2, Marseille, France).

Cycle thresholds were normalized to *rpoB* or *gyrB* levels and fold changes were calculated against the normalized control of induction and untreated values when applicable. The relative quantification of glycosyltransferase and gene expression was performed using the comparative ∆∆Ct method [45]. Each sample was treated in triplicate to ensure statistical significance of the analysis. The *P* value was determined by *t*-test. The significance *P* values shown are at least <0.005. As a control, additional reactions were performed using, as a template, a Reverse Transcription mixture without enzyme, a Reverse Transcription mixture without RNA and chromosomal DNA.

### *In silico* analysis

The gene model from locus RUGNEv3_61221 of *Ruminococcus gnavus* E1 (GenBank accession no. FQ790378.1) was downloaded from the Microbial Genome Annotation and Analysis Platform (MaGE, accessible via https://www.genoscope.cns.fr/) and translated to the corresponding protein sequence (GenBank accession no. CCA61958.1), herein referred to as *Rg*SPP. Eighteen hundred other amino acid sequences of bacterial GH13_18 members were extracted from the public version of the CAZy database [6]. The *Rg*SPP sequence was submitted to the Phyre2 server [46] in order to generate a 3D structural model and visualized using PyMOL software (PyMOL Molecular Graphics System, version 2.0; Schrödinger). The SignalP 4.1 server was used to determine the presence and location of protein signal peptide cleavage sites in the *Rg*SPP amino acid sequence [47]. Multiple alignment of characterized GH13_18 members was performed using mafft einsi advanced mode [48]. The secondary structure of AAO33821 from *Bifidobacterium adolescentis* based on its solved crystal structure (PDBID: 1R7A) was analysed using ESPript (http://espript.ibcp.fr) [49]. Where present, signal sequences and additional modules were removed to isolate the catalytic modules for bioinformatics analysis. A multiple sequence alignment was produced using the Muscle program [50], and the evolutionary history was inferred using the neighbour-joining method [51]. The evolutionary distances were computed using the Poisson correction method [52] and are in the following units: the number of amino acid substitutions per site. The analysis involved 1065 amino acid sequences. All ambiguous positions were removed for each sequence pair. There was a total of 987 positions in the final dataset. Evolutionary analyses were conducted in mega6 software [53].

Cluster analysis was based on the neighbour-joining method with the closely related bacterium *Ruminococcus gnavus* E1 as the out-group root. Synteny blocks were analysed by the MaGE platform, which allowed the comparison of coding sequencespredicted from the genomic DNA of *Ruminococcus gnavus* E1 to those predicted from genomic DNA present in the PkGDB (Prokaryotic Genome DataBase) and the National Center for Biotechnology Information RefSeq database (a collection of raw sequences from whole-genome sequencing). Beyond a simple sequence comparison, this interface allows the analysis of the synteny between two chromosomes.

### Gene prevalence and abundance analysis

Sequences of the GH13_18 referenced in the CAZy database in September 2018 were searched by blastp analysis (*E* value=0, identity ≥90 %) in the translated catalogue of 9.9 million reference genes constructed using gut metagenome sequences of 1267 subjects from 3 continents (USA, China, Europe): 139 USA HMP samples; 760 European faecal samples from the MetaHIT project; 368 Chinese faecal samples [54]. The microbial gene richness of GH13_18 in human gut was assessed by recovering the prevalence and occurrence frequency data [54] of homologous sequence of the catalogue assigned to GH13_18 from the 9.9 gene frequency matrix in the 1267 subjects (http://meta.genomics.cn/meta/dataTools).

## Results and Discussion

### Cloning, heterologous expression and functional characterization of *Rg*SPP

**Fig. 1.**
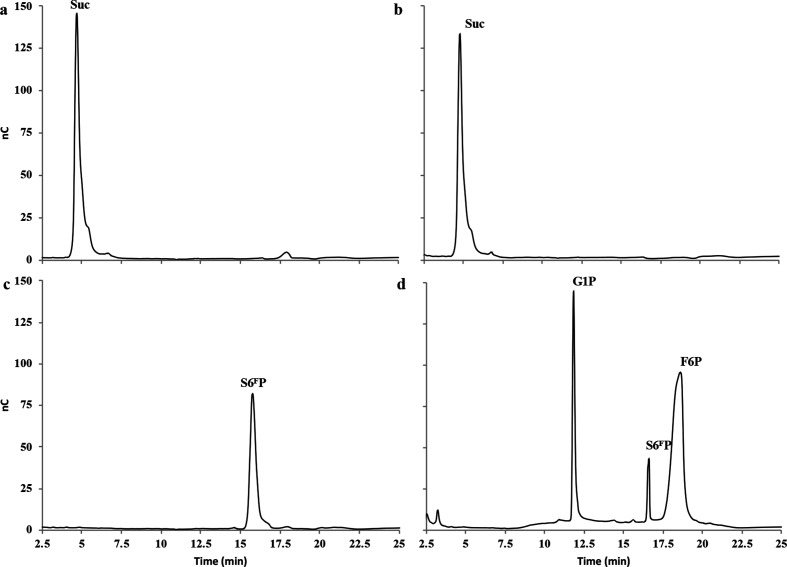
HPAEC-PAD profiles of the *Rg*SPP reaction products obtained after 24 h in the presence of 20 mM sucrose (b) and S6^F^P (d) and Pi (phosphorolysis). Controls without enzyme are displayed for sucrose (a) and S6^F^P (c).

**Fig. 2.**
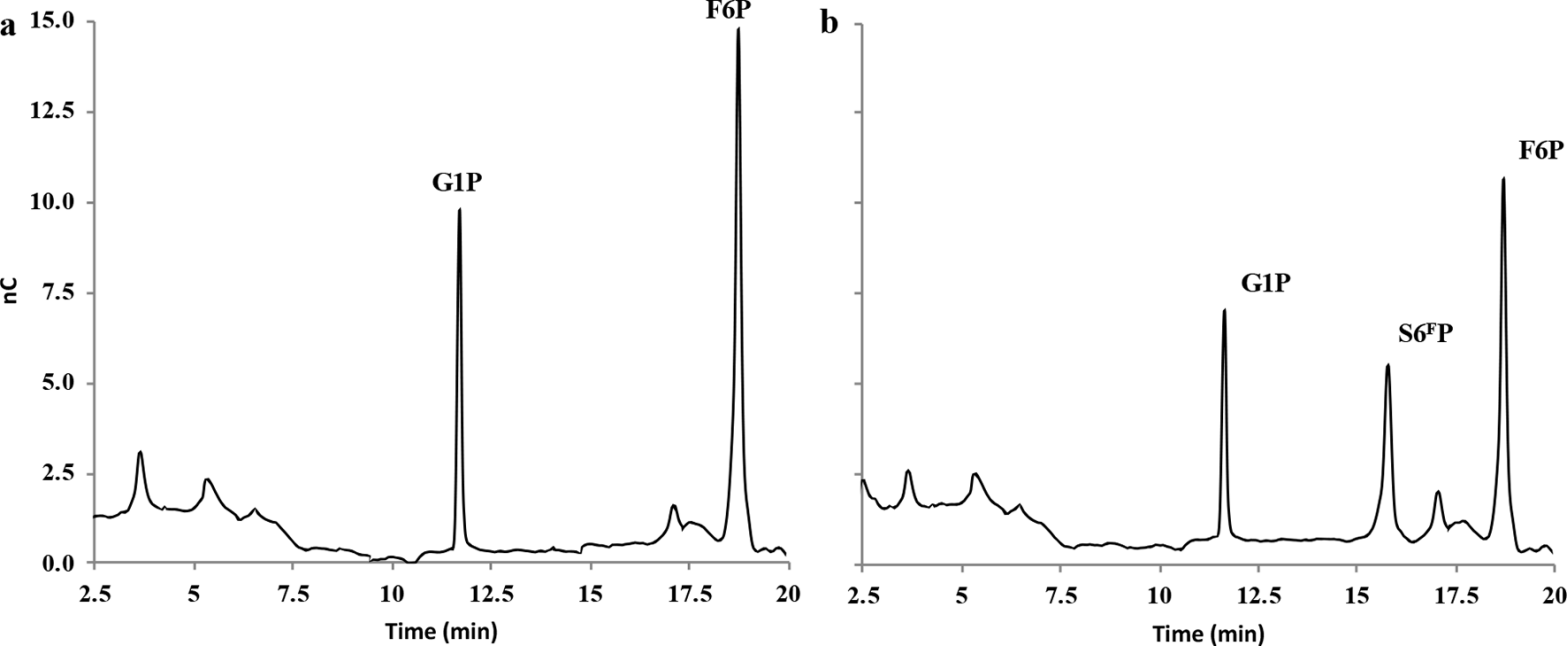
HPAEC-PAD profiles of the *Rg*SPP reaction products obtained after 24 h in the presence of 100 µM G1P and F6P (reverse phosphorolysis). (a) Negative control and (b) assay with 0.18 µM *Rg*SPP.

In order to perform biochemical and molecular studies, the *Rg*SPP-encoding gene was cloned into the pOPINE vector, and heterologously expressed in *E. coli* BL21. After affinity purification using immobilized metal affinity chromatography (Ni-NTA, Fig. S1a) and an additional SEC (S200; Fig. S1b), the recombinant protein tagged with a C-terminal (His)_6_-tag showed on SDS-PAGE an apparent molecular mass of 55 kDa (Fig. S2), which is in agreement with its theoretical molecular mass (56 165 Da). SEC analysis indicated that *Rg*SPP is a monomer in solution (Fig. S1b). The purified *Rg*SPP was first assayed against sucrose and S6^F^P in the presence of inorganic phosphate, in order to determine its phosphorolysis activity. G1P and F6P were released only from S6^F^P, indicating that the enzyme is a SPP and not a SP (Figs 1 and S3). In order to accurately determine the substrate specificity of *Rg*SPP, different carbohydrates and phosphorylated carbohydrates were then considered as acceptors for the reverse phosphorolysis reaction in the presence of G1P as glycosyl donor (Table 1). The results showed that the *Rg*SPP only used the F6P as acceptor. Considering this S6^F^P synthetic reaction (Fig. 2), *Rg*SPP displayed a specific activity of 0.22 U mg^−1^. All in all, these data obtained by HPAEC-PAD, demonstrate that *Rg*SPP is a highly specific enzyme, able to reversely convert S6^F^P+Pi into G1P+F6P (Figs 1 and 2). Previously, this activity was reported only once for a member of the GH13_18 family, from *Thermoanaerobacterium thermosaccharolyticum* (*Tt*SPP), which is a thermophilic obligate anaerobe [25]. However, SPP activity has never been described within the human gut microbiota so far.

**Table 1.** *Rg*SPP activity on various substrates

**Reaction**	**Substrate**	**Relative activity (%)**
Phosphorolysis*	Sucrose 6^F^-phosphate	100
	Sucrose	nd
Synthesis**†**	d-Fructose-6-phosphate	100
	d-Fructose	nd
	d-Glucose	nd
	d-Galactose	nd
	d-Mannose	nd
	d-*N*-Acetylgalactosamine	nd
	d-Xylose	nd
	Maltotetraose	nd
	Maltoheptaose	nd

nd, Not detected.

*At 40 °C with 100 mM phosphate buffer at pH 6.5 and 20 mM donor.

†At 40 °C with 50 mM MOPS buffer at pH 6.5, 10 mM donor (α-d-glucose 1-phosphate) and 10 mM acceptor.

The biochemical properties of *Rg*SPP were then investigated for S6^F^P phosphorolysis. The optimum pH was 6.0 (Fig. S4a). This value corresponds to that obtained with the other characterized members of the GH13_18 family (between 6.0 and 6.5), which are, as described further, all bacterial phosphorylases. This is in agreement with the physiological role of these enzymes, which is to perform intracellular phosphorolysis of oligosaccharides. The optimal temperature was 45 °C, a value surprisingly higher than that met in the human gut (Fig. S4b). Such thermoactive enzymes have already been found in the human gut microbiome, and could be due to the ability of gut microbes to colonize various habitats [55]. In addition, fridge storage cannot be considered because the purified protein was not stable at 4 °C after 3 days, whereas *Rg*SPP was stable for at least 4 weeks at −20 °C (data not shown). *Rg*SPP phosphorolysis *K*_m_ and *k_cat_* values are summarized in Table 2. This enzyme is 112 times more efficient for S6^F^P phosphorolysis than *Tt*SPP [25]. Moreover, it is noteworthy that *Rg*SPP is strictly specific for S6^F^P, in contrast to *Tt*SPP, which also displays high activity on sucrose.

**Table 2.** *Rg*SPP kinetic parameters determined on sucrose 6^F^-phosphate

**Reaction**	**Enzyme**	***K_m_*** **(mM)**	***k_cat_*** **(s^−1^)**	***k_cat_*/*K_m_*** **(mM^−1^ s^−1^)**	**Reference**
Phosphorolysis	*Rg*SPP	1.70	740	435.3	Present work
	*Tt*SPP	12.7	82.6	6.5	[23]

### Sequence analysis

Based on sequence alignment, *Rg*SPP belongs to glycoside hydrolase family GH13 subfamily 18 according to the CAZy classification (http://www.cazy.org/ [6]). SignalP failed to predict a signal peptide suggesting a cytoplasmic subcellular localization [47].

Multiple sequence alignment for all the characterized members of the GH13_18 family with *Rg*SPP was performed (Fig. S5). The deduced amino acid sequence (492 aa) was compared to other characterized SP, GGaP and GGoP sequences, and revealed about 32 to 35 % identities to the characterized SPs from *Leuconostoc mesenteroides* spp. (BAA14344.1 [17], ABS59292.1 [21], AAX33736.1 [23]), *Streptococcus mutans* (CAA30846.1 [16]), *Bifidobacterium adolescentis* (AAO33821.1 [20]), *Bifidobacterium longum* spp. (AAO84039.1 [19], BAF62433.1 [22]), *Lactobacillus reuteri* (AGK37834.1 [24]), *Pelomonas saccharophila* (AAD40317.1 [18]); 34 % to *Tt*SPP from *Thermoanaerobacterium thermosaccharolyticum* (ADL69407.1 [25]); 27 and 28 % to *Ms*GGaP and *Ma*GGoP (ADH62582.1 and ADP98617.1 [26, 27]), respectively (Fig. S5). Previously Verhaeghe *et al.*, in 2014, proposed that the residue H344 in *Tt*SPP is present in all SPP [25]. The mutation of this residue H344 to Tyr led to a decreased ratio of activity on phosphorylated fructose over fructose, suggesting a crucial role of this residue in phosphate binding. However, in *Rg*SPP, the equivalent residue for H344 in *Tt*SPP is a tyrosine (Y355), as observed for all the others characterized members of the subfamily, including the GGaPs and GGoPs, suggesting that the S6^F^P specificity might not be restricted to this residue. This observation is corroborated by the observation of Y344 – the homologous residue in SP from *Bifidobacterium adolescentis* (AAO33821, PDBID 1R7A) – which has a side chain pointing out of the overall structure is unlikely involved in enzyme activity [20]. Nevertheless, we can speculate that the hydroxyl function could form an H bond or even a phosphoester bridge with the phosphate carried by the C6 of the fructose residue to stabilize the substrate into the catalytic pocket.

### Phylogenetic, synteny analysis and metabolic pathways

**Fig. 3.**
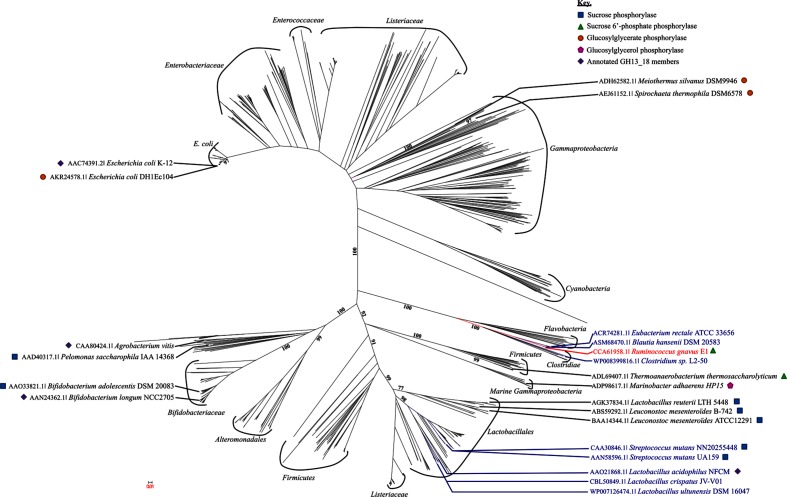
Phylogenic tree of subfamily GH13_18. The tree includes all the GH13_18 listed in the CAZy database. Members sharing syntenies with *Rgspp* are indicated in dark blue. Enzyme specificity is indicated in the key.

In order to gain more insight in the phylogenetic diversity of the GH13_18 subfamily, a phylogenetic tree was reconstructed, using sequences classified in the CAZy database (Fig. 3) [6]. GH13_18 enzymes are distributed in diverse phylogenetic groups, such as lactic acid bacteria or cyanobacteria from different ecosystems like soil, marine and human gastrointestinal microbiota. The present phylogenetic tree reveals different clades; seven containing characterized SP, SPP, GGaP or GGoP enzymes (e.g. *Bifidobacteriaceae*, *Lactobacilliales* and *Thermoanaerobacteria* [10, 25–27, 56]). Franceus *et al*., in 2017, proposed a phylogenetic tree of the GH13_18 subfamily with two major clusters, one with all SP and SPP enzymes and the other one with enzymes exhibiting GGaP activity, but this was without taking into account the identification of the new *Ma*GGoP that is interlocked between the SP and SPP enzymes [26, 27]. Consequently, GH13_18 activity cannot be predicted from its clade status. Here, we observed that *Rg*SPP belongs to the *Clostridiae* clade constituted by only gut bacteria (e.g. *Blautia*, *Clostridium*, *Eubacterium*) (Fig. 3) despite the fact that *Rg*SPP and *Tt*SPP, which shared the same substrate specificity, clustered in two different but close clades. It is noteworthy that most of the GH13_18 were found in the lactic acid bacteria group, known for their diverse benefits for humans (e.g. probiotic, oral health, etc.), underlying the importance of such activities.

**Fig. 4.**
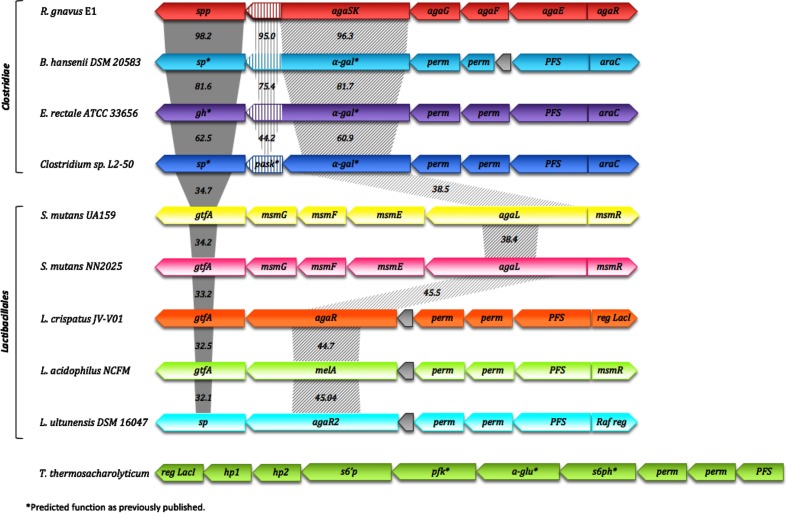
Genomic environment of the *Rgspp* gene. The values indicate the percentage identities between *spp* or *sp*, *kinase* and *α-gal* genes.

**Fig. 5.**
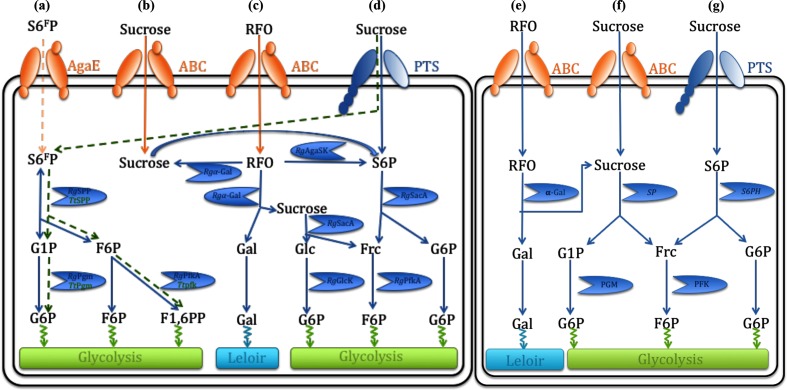
Hypothetical sucrose, sucrose 6-phosphate and sucrose 6^F^-phosphate metabolic pathways in the *Ruminococcus gnavus* E1, *T. thermolyticum* and *Lactobacillales* bacteria (adapted from Bruel *et al*. and Verhaeghe *et al.* [24, 32]). Pathways for uptake and catabolism in the *Ruminococcus gnavus* E1 clade of (a) S6^F^P, (b) and (d) sucrose, and (c) RFO, and in the lactobacillales bacteria of (e) RFO, and (f) and (g) sucrose. The *T. thermolyticum* S6F P (a) metabolic pathway proposed by Verhaeghe *et al.*, in 2014, appears in green, whereas the sucrose pathways follow the (f) and (g) models. *Rg*SPP, sucrose 6^F^-phosphorylase; *Rg*AgaSK, α-galactosidase/sucrose kinase; Rgα-Gal, α-galactosidase; *Rg*PfkA, 6-phosphofructokinase; *Rg*Pgm, phosphoglucomutase; *Rg*SacA, sucrose 6P hydrolase; *Rg*GlcK, glucose kinase; SP, sucrose phosphorylase; S6PH, sucrose 6-phospho-hydrolase; AgaE and ABC, ABC transporters; PTS, phosphoenolpyruvate-dependent sugar phosphotransferase system; S6^F^P, sucrose 6^F^-phosphate; S6P, sucrose 6-phosphate; RFO, raffinose family oligosaccharide.

As previously mentioned, the *Rgspp* gene is located in the *Rgaga1* locus previously described by Bruel *et al.*, in 2011 [33], of which the organization is similar to (i) the *Rgaga2* locus suggested to play essential roles in the metabolism of dietary sucrose and oligosaccharides [35], and (ii) polysaccharide utilization loci (PUL) as defined by Terrapon *et al.*, in 2015 [57], but with an ABC transporter rather than a TonB-dependent transporter/SusD family lipoprotein-encoding gene pair specific from the *Bacteroidetes* [58]. In order to figure out the accurate role of such Gram-positive PUL in the human gut microbiome, we performed a synteny study based on all the prokaryotic strains with characterized GH13_18 enzymes to date (Fig. 4), coupled with a deep mining of the *Ruminococcus gnavus* E1 genome, that led us to propose a new model of sucrose, S6^F^P and RFO metabolic pathways (Fig. 5a–g). The clustering alignment showed for the four first sequences found in the *Clostridiae* clade of the phylogenetic tree, a quite high conservation of this classical Gram-positive PUL organization [58], where *spp* genes are always in a position downstream of those encoding bi-functional enzymes presenting kinase and α-galactosidase activities. Moreover, the different ABC-transporter- and PTS-encoding genes (i.e. *agaG*, *agaF* and *agaE* for the *Ruminococcus gnavus* E1 cluster, and *perm* and *PFS* for *Blautia hansenii, Eubacterium rectale* and *Clostidium spL2-50*) and the response regulator genes (i.e. *araC*), located in a position upstream of the *kinase-α-gal* gene are also conserved. The comparison between these clusters showed that the *spp*, *kinase* and *α-gal* gene sequences are highly conserved, with at least 62.5, 44.2 and 60.9 % identities, respectively, whereas identities were less important with enzymes originally from the *Lactobacillale* clade (Fig. 4). Although, synteny is observed with clusters from different genomes belonging to the *Lactobacillale* clade where the *α-gal* gene is still conserved, the *kinase* gene is not. Interestingly, the absence of *kinase* genes in the *Lactobacillale* clusters appears to be correlated with the presence of a *sp* gene as described for the five GH13_18 characterized enzymes from *Streptococcus mutans*, *Lactobacillus crispatus*, *Lactobacillus acidophilus* and *Lactobacillus ultunensis* [59–62] suggestive of different sucrose and RFO metabolic pathways (Fig. 5). Indeed, the absence of a *kinase* gene led to (i) an inability to phosphorylate sucrose or RFOs to S6P contrary to the *Ruminococcus gnavus* E1 clade via SK-α-Gal enzymes (Figs. 4 and 5b–d) and (ii) confirmed the involvement of the PTS for the phosphorylation of sucrose in S6P, and the subsequent hydrolysis in Frc and G6P by a sucrose 6-phosphate hydrolase (see *Rg*SacA accession no. CCG93499.1; E.C. 3.2.1.26 and S6PH in Fig. 5d, g, respectively). Fructose is phosphorylated afterwards by a fructokinase to F6P (*Rg*Fruk, accession no. EDN78042.1; E.C. 2.7.1.11; Fig. 5d, g). Moreover, the conservation of the *α-gal* gene shows the ability to remove the galactosyl units from the RFOs, which then will be managed by the metabolic pathway of Leloir for the *Ruminococcus gnavus* E1 and *Lactobacillale* bacteria (Fig. 5c, e). Finally, considering the two clusters carrying the only two SPP characterized to date, i.e. *Rg*SPP and *Tt*SPP, we observed that surprisingly there is no synteny between the two organisms (Fig. 4). Nevertheless, it appeared that there is a real relationship between the SPP activity and the presence of a kinase catalytic domain in the same cluster (i.e. AgaSK in the *Rgaga1* cluster and PFK in the *Ttspp* cluster). Strengthening the previous hypothesis from Verhaeghe *et al.*, in 2014 [25], where sucrose is phosphorylated in S6^F^P during translocation by a PTS; here, we propose a translocation of the S6^F^P via the AgaE PTS component. Then, S6^F^P could be metabolized by *Rg*SPP to produce on one hand G1P, which is subsequently transformed by a phosphoglucomutase (*Rg*Pgm accession no. WP_101882627.1; E.C. 5.4.2.2) into G6P, on the other hand F6P could end up as intermediate in the glycolytic pathway or could be phosphorylated first by *Rg*Pfka yielding Fructose 1,6-di-phosphate (F1,6PP) (Fig. 5a).

Finally, no synteny has been identified between the *Rgaga1* cluster and the GGaP and GGoP genomic environments recently characterized by Franceus *et al.*, in 2017 and 2018, respectively [26, 27], suggesting that this PUL organization is linked to the sucrose, S6^F^P and RFO metabolic pathways [33, 35].

### Prevalence in the human gut microbiota

**Fig. 6.**
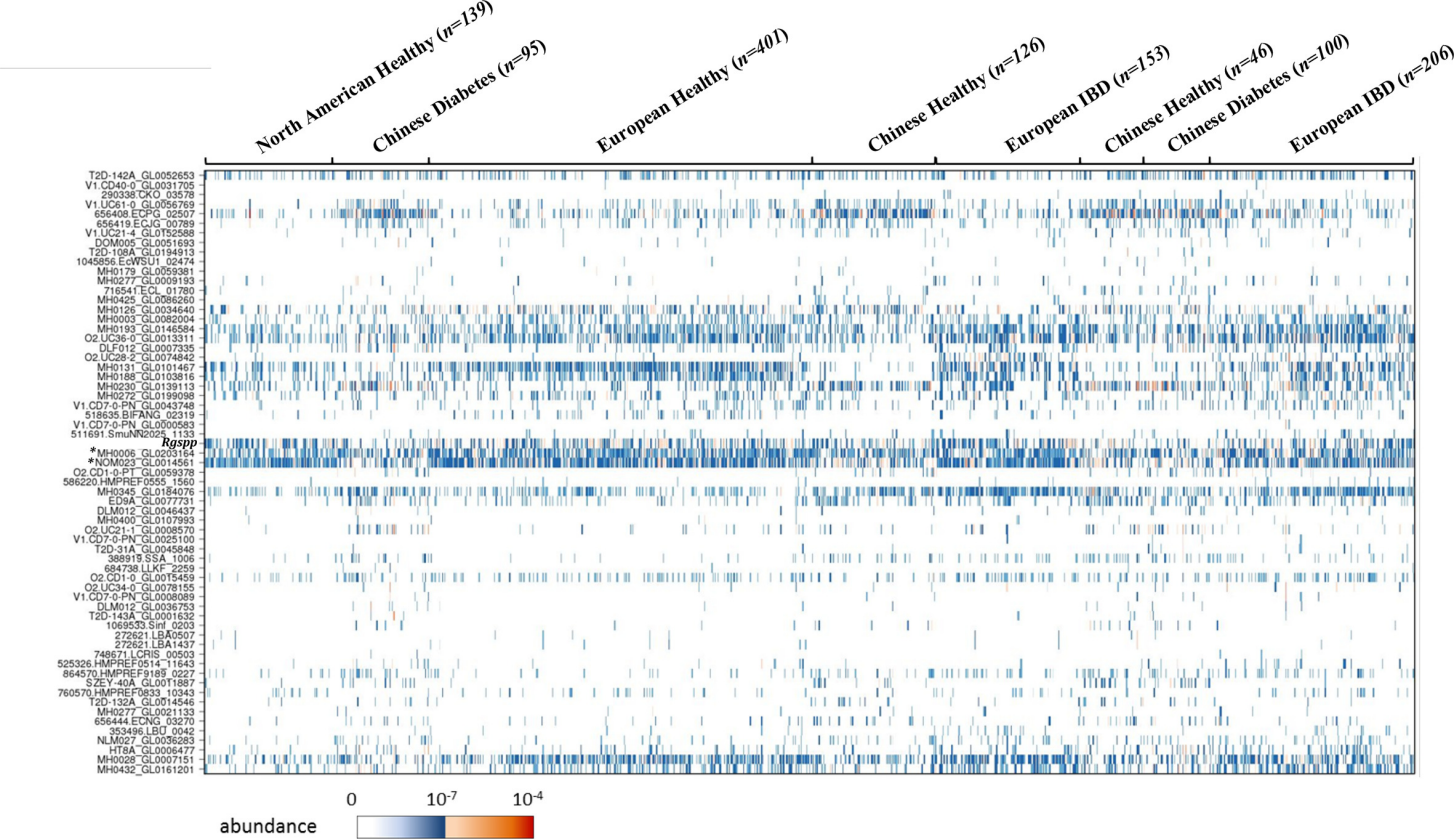
Prevalence and abundance of the human gut GH13_18 gene homologues found in the human gut reference catalogue (1267 individuals from diverse geographical origins and with different health status). Connections between GH13_18 sequences and sequences from the catalogue are indicated in Table S3. The target *Rgspp* shares, respectively, 97.8 and 98.4 % identities with the sequences MH0006_GL0203164 (*) and NOM023_GL0014561 (*) of the catalogue.

Among the GH13_18 sequences referenced in the CAZy database, 954 shared at least 90 % sequence identity with 63 sequences of the human gut metagenomic gene catalogue established from 1267 subjects [54]. Their abundance and prevalence in the various cohorts are presented in Fig. 6 and values are summarized in Table S3.

By investigating the prevalence of the GH13_18 homologues in individuals, we observed that there are differences due to geographical origin rather than an impact of the health status of the individuals. Indeed, the genes from *E. coli* strains corresponding to the sequences V1.UC61-0_GL0056769, 656408.ECPG_02507 and 656419.ECJG_00789 of the catalogue were more abundant in the Chinese subjects, together with the gene ADQ02266.1 (100 % identity with the sequence MH0230_GL0139113 of the catalogue) from the *Bifidobacterium longum* subsp. *longum* BBMN68 strain [63]. This strain, described as a health-promoting strain, was previously isolated from the faeces of a healthy Chinese centenarian. By contrast, other genes were less abundant in Chinese subjects, such as sequence AAO33821.1 from *Bifidobacterium adolescentis* DSM 20083 (99.8 % identity with MH0131_GL0101467), sequence CBL13462.1 from *Roseburia intestinalis* (97.6 % identity with 72-stool_revised_C812172_1_gene66964) and sequence ADC85157.1 from *Bifidobacterium animalis* subsp. *lactis* BB-12 (100 % identity with O2.UC28-2_GL0074842), independently of the clinical status. Besides, sequence AFJ26298.1 from *Streptococcus parasanguinis* (98.6 % identity with MH0003_GL0082004), sequence ACD98776.1 from *Bifidobacterium longum* DJO10A (99.6 % identity with MH0432_GL0161201) and to a lesser extent sequence AOL09592.1 from *Bifidobacterium longum* 35 664 (99 % identity with MH0193_GL0146584) were poorly represented in Chinese and USA subjects compared to European ones. It is noteworthy that only two sequences appear as biomarkers of the European subjects whose clinical status was intestinal bowel disease (IBD) [28]. One is the homologue sequence of ADC85157.1 from *Bifidobacterium animalis* subsp. *lactis* BB-12 (100 % identity with O2.UC28-2_GL0074842), which is in agreement with the study of Hao *et al.*, published in 2011, where the administration of *Bifidobacterium animalis* subsp. *lactis* BB-12 in combination with other probiotics to 32 patients with ulcerative colitis tended to increase remission [63, 64]. The other one, which is highly prevalent (found in 45 % of individuals), is the sequence CED94009.1 from *Romboutsia ilealis* CRIB (98.8 % identity with MH0345_GL0184076). These results provide new insights about the presence of homologous sequences from *Romboutsia ilealis* related to IBD. Indeed, Gerritsen *et al.* were not able to draw conclusions about the correlations between prevalence and/or abundance of *Romboutsia ilealis* and specific human diseases likely due to the limited number of available human datasets [65].

The target *Rg*SPP sequence and its homologues from *Eubacterium rectale* (ACR74281.1) present about 98 % identity with the sequences MH0006_GL0203164 and NOM023_GL0014561 of the catalogue. Both sequences are the most prevalent genes of the family and were found in 67.9 and 72.1 % of the individuals, respectively. They are distributed in the various cohorts, to a lesser extent within the Chinese cohort, whatever their clinical status. Similarly, the homologous sequences from *Blautia* (ASM68470.1, 97.8 % identity with MH0028_GL0007151) and *Clostridium* (WP_008399816, 98.4 % identity with MH0126_GL0034640) present a relatively high prevalence and are found in 50.9 and 29.7 % of individuals, respectively. Moreover, the sequence AAO33821.1 from *Bifidobacterium adolescentis* DSM 20083 (99.8 % identity with MH0131_GL0101467), encoding a characterized SP [20], presents a prevalence of about 45.5 % with a specific geographical distribution among European and USA cohorts independently of the clinical status. The other homologous sequences known to encode characterized SPs, whose homologues were also associated within the catalogue sequences, were found in less than 5 % of the individuals. Interestingly, the sequence AKR24578.1 from *E. coli* DH1Ec104 (99.4 % identity with 656408.ECPG_02507), which was characterized as a GGaP [26], is present in about 42.4 % of the individuals with a clear abundance in the Chinese cohorts independently of the clinical status. In total, only 17 sequences were found in more than 20 % of the subjects. For most of them it was possible to discriminate the geographical origin of the individuals, with differential abundances in the Chinese cohorts compared to the European and/or USA ones, suggesting that these differences are not related to the clinical status of the individuals but most likely to diet, indicative of the presence of sucrose-related compounds in their food intakes.

### HF diet influence on *Rgspp* mRNA expression in monoxenic mice

**Fig. 7.**
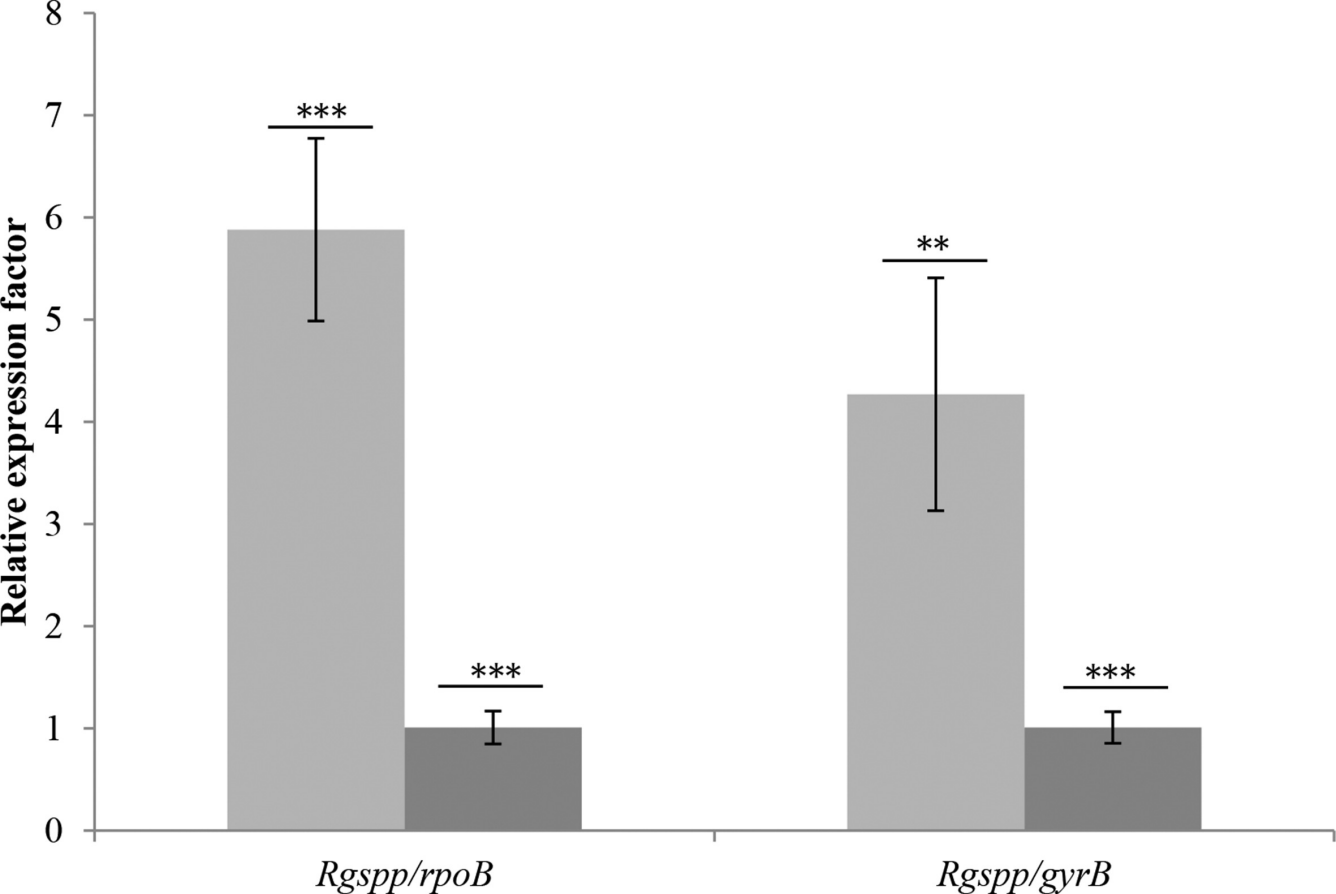
Expression of the RgSPP-encoding gene in monoxenic mice fed with different diets. Dark grey, SD (*n*=10); light grey, HF diet (*n*=10). Two different housekeeping genes were used to normalize the experiment, *rpoB* (left) and *gyrB* (right). *P* values were determined using Student's *t*-test. **, *P*<0.005; ***, *P*<0.001.

Although the abundance of the *Rgspp* sequence in the gut microbiome was not found specifically correlated to IBD or diabetes status but mostly to diet, we wanted to check whether diet could affect the expression of the *Rgspp* gene and, more globally, carbohydrate metabolism. It is indeed well known that, for example, a HF diet affects the composition of the gut microbiota, together with the host metabolism [66]. However, the influence of this diet on bacterial metabolism is poorly studied. In 2014, Daniel *et al*. fed mice with HF diet for 12 weeks [67]. Metaproteomic and metabolomic data obtained, targeting 1760 bacterial proteins and 86 annotated metabolites, revealed distinct HF-diet-specific profiles demonstrating the impact of diet on the microbial metabolism. In the present study, we used two different trials composed of monoxenic mice inoculated with *Ruminococcus gnavus* E1. In trial one, mice were fed with a SD and in trial two with a HF diet (Table S2), which contained five times less sucrose. As shown in Fig. 7, a fivefold increase in *Rgspp* mRNA was observed for the HF diet group, suggesting a close relationship between lipid and sucrose metabolism, and confirming the impact of the HF diet on the gut microbiome previously highlighted by Daniel *et al*. in 2014 [67].

### Conclusion

It is well-know that in plants the biosynthesis of sucrose occurs in the cytosol of the leaves, the two last steps being: (i) UDP-glucose+fructose-6*P*<>sucrose 6^F^-phosphate+UDP catalysed by a sucrose phosphate synthase; and (ii) sucrose 6^F^-phosphate+H_2_O<>sucrose+Pi catalysed by a SPP. We can assume that during consumption of plant dietary constituents, a part of S6^F^P is ingested, reaching the distal intestinal compartment without any modification (Fig. 5a). At this point, we can suggest a translocation of the S6^F^P across the *Ruminococcus gnavus* E1 cell wall to the cytosolic compartment, via the ABC transporter AgaE. S6^F^P will be then hydrolyses by *Rg*SPP releasing G1P and F6P, considered as metabolic intermediates of microbial carbohydrate catabolism. Nevertheless, more investigation looks to be necessary to confirm these hypotheses.

## Data Bibliography

1. Additional sequences and data used in this study were obtained from the following sources.
2. Verhaeghe T, Aerts D, Diricks M, Soetaert W, Desmet T. The quest for a thermostable sucrose phosphorylase reveals sucrose 6’-phosphate phosphorylase as a novel specificity. *Appl Microbiol Biotechnol* 2014;98:7027–7037.
3. Bruel L, Sulzenbacher G, Cervera Tison M, Pujol A, Nicoletti C, Perrier J *et al*. α-Galactosidase/sucrose kinase (AgaSK), a novel bifunctional enzyme from the human microbiome coupling galactosidase and kinase activities. *J Biol Chem* 2011;286:40814–40823.
4. Franceus J, Pinel D, Desmet T. Glucosylglycerate phosphorylase, an enzyme with novel specificity involved in compatible solute metabolism. *Appl Environ Microbiol* 2017;83:e01434-17.
5. Franceus J, Decuyper L, D’Hooghe M, Desmet T. Exploring the sequence diversity in glycoside hydrolase family 13_18 reveals a novel glucosylglycerol phosphorylase. *Appl Microbiol Biotechnol* 2018;102:3183–3191.
6. Qin J, Li R, Raes J, Arumugam M, Burgdorf KS *et al*. A human gut microbial gene catalogue established by metagenomic sequencing. *Nature* 2010;464:59–65.

## Supplementary Data

Supplementary File 1
