# Supplementary material for: Sucrose 6F-phosphate phosphorylase: a novel insight in the human gut microbiome
Source: Microb Genom. 2019 Mar 26;5(4):e000253. doi: 10.1099/mgen.0.000253 (PMC6521584; doi:10.1099/mgen.0.000253)
Supplement: Supplementary File 1 [file mgen-5-253-s001.pdf]

Supplementary information

Table S1. Primer used during PCR and qRT-PCR assays

	Primers name	Primers sequence 5' – 3'
**PCR**	*Rgspp-F*	TCACCAAAAGTGTGGGAGTTT
	Rgspp-R	CTGCGCCGTAAGAGGCTAT
**qRT-PCR**	*Rgspp-F*	AGGAGATATACCATGAACAGTAAACCGATG
	Rgspp-R	GTGATGGTGATGTTTTTTGCTTTCTACTAT
	RgrpoB-F	GGGGAAGAACTGGAGAACGTA
	RgrpoB-R	TTCCATATCAGGAGCGAAATC
	RggyrB-F	CAATTCTGTGACGGTCGTTG
	RggyrB-R	AATCCCTGCCTTGTGGTTTA
**16S RNA**	*16F8*	AGAGTTTGATCCTGGCTGAG
	16R1541	AAGGAGGTGATCCAGCCGCA

Primers sequences used for the cloning of *Rgspp* and for qRT-PCR and 16S RNA analysis.

Table S2. Composition of the diets used for *in vivo* assays

Ingredient, g/kg (as-fed basis)	Control diet	High Fat diet
Casein	200	200
L- Cystine	3	3
Maltodextrin	320	125
Sucrose	350	68.8
scFOS	0	0
Cellulose	50	50
Soybean oil	25	25
Lard	20	245
Mineral mix[1]	10	10
Analyzed Composition (DM basis)		
DM[2], g/100g		
Crude Protein, g/100g	19.2	26.2
Carbohydrates, g/100g	67.3	26.3
Fat[2], g/100g	4.3	34.9
Energy, kcal/kg	3.84	5.24

[1]Di-calcium phosphate, calcium carbonate, potassium citrate, vitamin mix, choline bitartrate, FD&C

Table S3. BlastP of GH13_18 sequences against the IGC.pep catalogue (E-value=0, identity ≥90%). For each sequence, the catalogue sequence presenting the highest identity value was kept. Among the GH13_18 sequences, 954 sequences showed homology with 64 sequences in the catalogue. *Rg*SPP, some characterized GH13_18 and the ones presenting high synteny with *Rg*SPP are indicated in bold.

Catalogue ID	Genbank accession number	Organism	% of identity	e-value	Number of subjects among the 1267	Prevalence (%)	Abundance
T2D-142A_GL0052653	AAN80246.1	Escherichia coli CFT073	97.80	0.0	389	30.70	0.00017529
T2D-142A_GL0052653	ABE07057.1	Escherichia coli UTI89	97.61	0.0			
T2D-142A_GL0052653	ABG69372.1	Escherichia coli 536	97.98	0.0			
T2D-142A_GL0052653	ABJ00761.1	Escherichia coli APEC O1	97.61	0.0			
T2D-142A_GL0052653	ADE89798.1	Escherichia coli IHE3034	97.44	0.0			
T2D-142A_GL0052653	ADN46145.1	Escherichia coli ABU 83972	97.80	0.0			
T2D-142A_GL0052653	ADN71421.1	Escherichia coli UM146	97.44	0.0			
T2D-142A_GL0052653	ADR26772.1	Escherichia coli O83:H1 str. NRG 857C	97.99	0.0			
T2D-142A_GL0052653	AEG36324.1	Escherichia coli NA114	97.80	0.0			
T2D-142A_GL0052653	AER84181.1	Escherichia coli str. clone D i2	97.80	0.0			
T2D-142A_GL0052653	AER89100.1	Escherichia coli str. clone D i14	97.80	0.0			
T2D-142A_GL0052653	AGY87305.1	Escherichia coli JJ1886	97.62	0.0			
T2D-142A_GL0052653	AID78459.1	Escherichia coli Nissle 1917	97.62	0.0			
T2D-142A_GL0052653	AIL16739.1	Escherichia coli ATCC 25922	97.62	0.0			
T2D-142A_GL0052653	AJB39624.1	Escherichia coli APEC IMT5155	97.44	0.0			
T2D-142A_GL0052653	AJM73548.1	Escherichia coli RS218	97.44	0.0			
T2D-142A_GL0052653	AJO83374.1	Escherichia coli MNCRE44	97.62	0.0			
T2D-142A_GL0052653	AKK42380.1	Escherichia coli NMEC O18	97.44	0.0			
T2D-142A_GL0052653	ALD25449.1	Escherichia coli SF-468	97.43	0.0			
T2D-142A_GL0052653	ALD30522.1	Escherichia coli SF-173	97.44	0.0			
T2D-142A_GL0052653	ALD35474.1	Escherichia coli SF-166	97.44	0.0			
T2D-142A_GL0052653	ALD40227.1	Escherichia coli SF-088	97.44	0.0			
T2D-142A_GL0052653	ALT49205.1	Escherichia coli uk_P46212	97.62	0.0			
T2D-142A_GL0052653	ALX52262.1	Escherichia coli CD306	97.62	0.0			
T2D-142A_GL0052653	ALX57369.1	Escherichia coli JJ2434	97.62	0.0			
T2D-142A_GL0052653	ALX62404.1	Escherichia coli JJ1897	97.62	0.0			
T2D-142A_GL0052653	AML04473.1	Escherichia coli G749	97.62	0.0			
T2D-142A_GL0052653	AML09432.1	Escherichia coli MVAST0167	97.62	0.0			
T2D-142A_GL0052653	AML14237.1	Escherichia coli SaT040	97.62	0.0			
T2D-142A_GL0052653	AML19255.1	Escherichia coli ZH193	97.62	0.0			
T2D-142A_GL0052653	AMM36204.1	Escherichia coli ZH063	97.44	0.0			
T2D-142A_GL0052653	AMQ51066.1	Escherichia coli JJ1887	97.62	0.0			
T2D-142A_GL0052653	AMX15579.1	Escherichia coli Ecol_732	97.62	0.0			
T2D-142A_GL0052653	AMX28949.1	Escherichia coli Ecol_743	97.62	0.0			
T2D-142A_GL0052653	AMX36582.1	Escherichia coli Ecol_745	97.62	0.0			
T2D-142A_GL0052653	AMX39419.1	Escherichia coli Ecol_448	97.62	0.0			
T2D-142A_GL0052653	ANJ42350.1	Escherichia coli NGF1	97.99	0.0			
T2D-142A_GL0052653	ANK01347.1	Escherichia coli O25b:H4	97.80	0.0			
T2D-142A_GL0052653	ANK32050.1	Escherichia coli ECONIH2	97.80	0.0			
T2D-142A_GL0052653	ANK53034.1	Escherichia coli Eco889	97.62	0.0			
T2D-142A_GL0052653	ANR85697.1	Escherichia coli K-15KW01	97.80	0.0			
T2D-142A_GL0052653	ANV93451.1	Escherichia coli UPEC 26-1	97.62	0.0			
T2D-142A_GL0052653	APK33892.1	Escherichia coli D8	97.25	0.0			
T2D-142A_GL0052653	AQV23300.1	Escherichia coli Ecol_276	97.62	0.0			
T2D-142A_GL0052653	AQV45525.1	Escherichia coli Ecol_542	97.62	0.0			
T2D-142A_GL0052653	AQV59457.1	Escherichia coli Ecol_656	97.62	0.0			
T2D-142A_GL0052653	AQV61545.1	Escherichia coli Ecol_867	97.62	0.0			
T2D-142A_GL0052653	AQV71396.1	Escherichia coli Ecol_AZ146	97.62	0.0			
T2D-142A_GL0052653	AQV82835.1	Escherichia coli Ecol_AZ153	97.62	0.0			
T2D-142A_GL0052653	AQW02399.1	Escherichia coli Ecol_AZ159	97.62	0.0			
T2D-142A_GL0052653	AQW09527.1	Escherichia coli Ecol_AZ161	97.62	0.0			
T2D-142A_GL0052653	AQW14315.1	Escherichia coli Ecol_AZ162	97.62	0.0			
T2D-142A_GL0052653	AQW17674.1	Escherichia coli Ecol_244	97.62	0.0			
T2D-142A_GL0052653	AQX96554.1	Escherichia coli NU14	97.44	0.0			
T2D-142A_GL0052653	ARA64169.1	Escherichia coli AR_0104	97.62	0.0			
T2D-142A_GL0052653	ARR63777.1	Escherichia coli strain Z247	97.62	0.0			
T2D-142A_GL0052653	ART44327.1	Escherichia coli 81009	97.62	0.0			
T2D-142A_GL0052653	ARX26029.1	Escherichia coli AR_0058	97.62	0.0			
T2D-142A_GL0052653	ASA42985.1	Escherichia coli H105	97.62	0.0			
T2D-142A_GL0052653	ASC19280.1	Escherichia coli AR_0055	97.62	0.0			
T2D-142A_GL0052653	ASJ30916.1	Escherichia coli PSUO2	97.99	0.0			
T2D-142A_GL0052653	ASW59453.1	Escherichia coli PA45B	97.44	0.0			
T2D-142A_GL0052653	ATB78588.1	Escherichia coli 317	97.99	0.0			
T2D-142A_GL0052653	ATC17600.1	Escherichia coli 1105	97.80	0.0			
T2D-142A_GL0052653	ATV08513.1	Escherichia coli BH100	97.80	0.0			
T2D-142A_GL0052653	ATV51204.1	Escherichia coli LS4	97.62	0.0			
T2D-142A_GL0052653	ATY17967.1	Escherichia coli AR_0017	97.99	0.0			
T2D-142A_GL0052653	AUF90477.1	Escherichia coli BH100	97.80	0.0			
T2D-142A_GL0052653	AUG64391.1	Escherichia coli ExPEC XM	97.44	0.0			
T2D-142A_GL0052653	AUG92998.1	Escherichia coli MS8345	97.25	0.0			
T2D-142A_GL0052653	AUM08401.1	Escherichia coli STEC299	97.44	0.0			
T2D-142A_GL0052653	AUN89926.1	Escherichia coli BH100N	97.80	0.0			
T2D-142A_GL0052653	AUQ36916.1	Escherichia coli BH100L	97.80	0.0			
T2D-142A_GL0052653	AUY42350.1	Escherichia coli HS13-1	97.44	0.0			
T2D-142A_GL0052653	AUZ93285.1	Escherichia coli FHI_NMBU_03	97.25	0.0			
T2D-142A_GL0052653	AVF98922.1	Escherichia coli FDAARGOS_144	97.62	0.0			
T2D-142A_GL0052653	AVN39919.1	Escherichia coli AR_0081	97.62	0.0			
T2D-142A_GL0052653	AVQ75855.1	Escherichia coli MS7163	97.44	0.0			
T2D-142A_GL0052653	AVV70792.1	Escherichia coli RM14723	97.80	0.0			
T2D-142A_GL0052653	AVV75339.1	Escherichia coli RM14715	97.80	0.0			
T2D-142A_GL0052653	AVZ59281.1	Escherichia coli Mt1B1	97.99	0.0			
T2D-142A_GL0052653	AWR74182.1	Escherichia coli E41-1	97.62	0.0			
T2D-142A_GL0052653	AWZ79519.1	Escherichia coli AR_451	97.62	0.0			
T2D-142A_GL0052653	BAI54830.1	Escherichia coli SE15	97.80	0.0			
T2D-142A_GL0052653	CAP75853.1	Escherichia coli LF82	97.99	0.0			
T2D-142A_GL0052653	CAR02770.1	Escherichia coli S88	97.44	0.0			
T2D-142A_GL0052653	CAR07715.2	Escherichia coli ED1a	97.44	0.0			
T2D-142A_GL0052653	CDH64955.1	Escherichia coli PMV-1	97.44	0.0			

T2D-142A_GL0052653	CDN81921.1	Escherichia coli O25b:H4-ST131 EC958	97.80	0.0			
T2D-142A_GL0052653	CKI13066.1	Escherichia coli NCTC9001	97.44	0.0			
V1.CD40-0_GL0031705	BAQ30221.1	Bifidobacterium scardovii JCM 12489 = DSM 13734	99.63	0.0	5	0.39	2.62754e-06
290338.CKO_03578	ABV14657.1	Citrobacter koseri ATCC BAA-895	100.00	0.0	17	1.34	1.38639e-06
290338.CKO_03578	ASE82016.1	Citrobacter koseri FDAARGOS_287	98.95	0.0			
290338.CKO_03578	ATF95846.1	Citrobacter koseri FDAARGOS_393	98.95	0.0			
290338.CKO_03578	AVE57097.1	Citrobacter koseri AR_0025	98.59	0.0			
290338.CKO_03578	AVE67039.1	Citrobacter koseri AR_0024	98.95	0.0			
290338.CKO_03578	CDZ85138.1	Citrobacter koseri	98.42	0.0			
V1.UC61-0_GL0056769	ACB15767.1	Escherichia coli SMS-3-5	99.82	0.0	256	20.21	0.000162746
V1.UC61-0_GL0056769	AEQ12362.1	Escherichia coli O7:K1 str. CE10	100.00	0.0			
V1.UC61-0_GL0056769	AIX63173.1	Escherichia coli ECONIH1	99.46	0.0			
V1.UC61-0_GL0056769	ALV68816.1	Escherichia coli ST648	99.46	0.0			
V1.UC61-0_GL0056769	AOM69735.1	Escherichia coli MS6198	99.46	0.0			
V1.UC61-0_GL0056769	AQV66632.1	Escherichia coli Ecol_881	99.46	0.0			
V1.UC61-0_GL0056769	ASI16718.1	Escherichia coli M160133	97.67	0.0			
V1.UC61-0_GL0056769	ASO87834.1	Escherichia coli PAR	95.71	0.0			
V1.UC61-0_GL0056769	ATB93258.1	Escherichia coli 1428	99.46	0.0			
V1.UC61-0_GL0056769	ATX15008.1	Escherichia coli CREC-544	98.57	0.0			
V1.UC61-0_GL0056769	AVJ13122.1	Escherichia coli WB61	97.67	0.0			
V1.UC61-0_GL0056769	AWR49132.1	Escherichia coli CCUG 70745	99.46	0.0			
V1.UC61-0_GL0056769	AWR84087.1	Escherichia coli H17	99.82	0.0			
V1.UC61-0_GL0056769	AWT01653.1	Escherichia coli 2016C-3878	98.57	0.0			
V1.UC61-0_GL0056769	AXG61473.1	Escherichia coli MCJCHV-1	95.71	0.0			
V1.UC61-0_GL0056769	BAX16129.1	Escherichia coli MRY15-117	98.57	0.0			
V1.UC61-0_GL0056769	BAX21068.1	Escherichia coli MRY15-131	98.57	0.0			
V1.UC61-0_GL0056769	CAR17791.1	Escherichia coli IAI39	99.82	0.0			
V1.UC61-0_GL0056769	CAS09049.1	Escherichia coli O127:H6 str. E2348/69	97.50	0.0			
V1.UC61-0_GL0056769	SLM06486.1	Escherichia coli O127:H6	97.50	0.0			
V1.UC61-0_GL0056769	SNU21699.1	Escherichia coli O127:H6	97.50	0.0			
656408.ECPG_02507	AAC74391.2	Escherichia coli str. K-12 substr. MG1655	99.64	0.0	537	42.38	0.00126729
656408.ECPG_02507	ABV05755.1	Escherichia coli HS	99.46	0.0			
656408.ECPG_02507	ABV18679.1	Escherichia coli O139:H28 str. E24377A	99.82	0.0			
656408.ECPG_02507	ACA77951.1	Escherichia coli ATCC 8739	99.82	0.0			
656408.ECPG_02507	ACB02530.1	Escherichia coli str. K-12 substr. DH10B	99.64	0.0			
656408.ECPG_02507	ACR64429.1	Escherichia coli BW2952 K-12	99.64	0.0			
656408.ECPG_02507	ACT29335.1	Escherichia coli BL21-Gold(DE3)pLysS AG	99.82	0.0			
656408.ECPG_02507	ACT38961.1	Escherichia coli B str. REL606	99.82	0.0			
656408.ECPG_02507	ACX39980.1	Escherichia coli DH1	99.64	0.0			
656408.ECPG_02507	ADT74891.1	Escherichia coli W	99.64	0.0			
656408.ECPG_02507	ADX51143.1	Escherichia coli KO11FL	99.64	0.0			
656408.ECPG_02507	AEE56242.1	Escherichia coli UMNK88	99.46	0.0			
656408.ECPG_02507	AEJ56318.1	Escherichia coli UMNF18	99.41	0.0			
656408.ECPG_02507	AFG40636.1	Escherichia coli P12b	99.65	0.0			
656408.ECPG_02507	AFS57432.1	Escherichia coli O104:H4 str. 2009EL-2050	99.82	0.0			
656408.ECPG_02507	AFS74642.1	Escherichia coli O104:H4 str. 2011C-3493	99.82	0.0			
656408.ECPG_02507	AFS86125.1	Escherichia coli O104:H4 str. 2009EL-2071	99.82	0.0			
656408.ECPG_02507	AGC86813.1	Escherichia coli APEC O78	99.82	0.0			
656408.ECPG_02507	AGW11524.1	Escherichia coli LY180	99.64	0.0			
656408.ECPG_02507	AGX33423.1	synthetic Escherichia coli C321.deltaA	99.64	0.0			
656408.ECPG_02507	AHM34759.1	Escherichia coli ST540	99.46	0.0			
656408.ECPG_02507	AIF36622.1	Escherichia coli KLY	99.64	0.0			
656408.ECPG_02507	AIF60392.1	Escherichia coli B7A	99.60	0.0			
656408.ECPG_02507	AIF60393.1	Escherichia coli B7A	99.66	0.0			
656408.ECPG_02507	AIN31770.1	Escherichia coli BW25113 K-12	99.64	0.0			
656408.ECPG_02507	AIT34502.1	Escherichia coli FAP1	100.00	0.0			
656408.ECPG_02507	AIZ27791.1	Escherichia coli ER2796	99.64	0.0			
656408.ECPG_02507	AIZ51116.1	Escherichia coli K-12 ER3413	99.64	0.0			
656408.ECPG_02507	AIZ82403.1	Escherichia coli RM9387	99.28	0.0			
656408.ECPG_02507	AIZ86885.1	Escherichia coli 94-3024	99.82	0.0			
656408.ECPG_02507	AJB51432.1	Escherichia coli 6409	99.82	0.0			
656408.ECPG_02507	AJE55666.1	Escherichia coli 789	99.82	0.0			
656408.ECPG_02507	AJF56182.1	Escherichia coli 1303	99.82	0.0			
656408.ECPG_02507	AJF76654.1	Escherichia coli C41(DE3)	99.82	0.0			
656408.ECPG_02507	AJG08293.1	Escherichia coli ECC-1470	99.82	0.0			
656408.ECPG_02507	AJH10173.1	Escherichia coli BL21 (TaKaRa)	99.82	0.0			
656408.ECPG_02507	AKA90383.1	Escherichia coli VR50	99.82	0.0			
656408.ECPG_02507	AKC12089.1	Escherichia coli CI5	99.82	0.0			
656408.ECPG_02507	AKD60861.1	Escherichia coli K-12 ER3454	99.64	0.0			
656408.ECPG_02507	AKD65235.1	Escherichia coli K-12 ER3440	99.64	0.0			
656408.ECPG_02507	AKD69604.1	Escherichia coli K-12 ER3476	99.64	0.0			
656408.ECPG_02507	AKD73956.1	Escherichia coli K-12 ER3445	99.64	0.0			
656408.ECPG_02507	AKD78366.1	Escherichia coli K-12 ER3466	99.64	0.0			
656408.ECPG_02507	AKD82735.1	Escherichia coli K-12 ER3446	99.64	0.0			
656408.ECPG_02507	AKD87095.1	Escherichia coli K-12 ER3475	99.64	0.0			
656408.ECPG_02507	AKD91505.1	Escherichia coli K-12 ER3435	99.64	0.0			
656408.ECPG_02507	AKE84592.1	Escherichia coli O104:H4 str. C227-11	99.82	0.0			
656408.ECPG_02507	AKF20376.1	Escherichia coli SEC470	99.64	0.0			
656408.ECPG_02507	AKF55202.1	Escherichia coli SQ37	99.64	0.0			
656408.ECPG_02507	AKF59342.1	Escherichia coli SQ88	99.64	0.0			
656408.ECPG_02507	AKF63480.1	Escherichia coli SQ110	99.64	0.0			
656408.ECPG_02507	AKF67620.1	Escherichia coli SQ171	99.64	0.0			
656408.ECPG_02507	AKF71760.1	Escherichia coli SQ2203	99.64	0.0			
656408.ECPG_02507	AKH26758.1	Escherichia coli CFSAN029787	99.82	0.0			
656408.ECPG_02507	AKK12937.1	Escherichia coli GM4792	99.64	0.0			
656408.ECPG_02507	AKK17468.1	Escherichia coli GM4792	99.64	0.0			
656408.ECPG_02507	AKN47429.1	Escherichia coli C43(DE3)	99.82	0.0			
656408.ECPG_02507	AKO56862.1	Escherichia coli NCM3722	99.64	0.0			
656408.ECPG_02507	AKP84048.1	Escherichia coli ACN001	99.82	0.0			
656408.ECPG_02507	AKR20224.1	Escherichia coli DH1Ec095	99.64	0.0			
**656408.ECPG_02507**	**AKR24578.1**	**Escherichia coli DH1Ec104**	**99.64**	**0.0**			
656408.ECPG_02507	AKR28981.1	Escherichia coli DH1Ec169	99.64	0.0			
656408.ECPG_02507	ALB31471.1	Escherichia coli RR1	99.64	0.0			
656408.ECPG_02507	ALI45309.1	Escherichia coli K-12	99.64	0.0			
656408.ECPG_02507	ALL93591.1	Escherichia coli 2012C-4227	99.64	0.0			
656408.ECPG_02507	ALN45422.1	Escherichia coli YD786	99.82	0.0			
656408.ECPG_02507	ALQ59522.1	Escherichia coli RS76	99.82	0.0			
656408.ECPG_02507	ALQ72912.1	Escherichia coli CQSW20	99.64	0.0			
656408.ECPG_02507	ALY13167.1	Escherichia coli ACN002	99.82	0.0			
656408.ECPG_02507	ALZ55843.1	Shigella sonnei FORC_011	98.84	0.0			
656408.ECPG_02507	AMB54552.1	Escherichia coli MRE600	97.85	0.0			
656408.ECPG_02507	AMH22195.1	Escherichia coli B C2566	99.82	0.0			
656408.ECPG_02507	AMH26506.1	Escherichia coli B C3029	99.82	0.0			
656408.ECPG_02507	AMH30021.1	Escherichia coli K-12 DHB4	99.64	0.0			
656408.ECPG_02507	AMH34744.1	Escherichia coli K-12 C3026	99.64	0.0			
656408.ECPG_02507	AML02098.1	Escherichia coli str. K-12 substr. MG1655 JW5437-1	99.64	0.0			
656408.ECPG_02507	AMR23057.1	Shigella sp. PAMC 28760	99.82	0.0			
656408.ECPG_02507	AMU81991.1	Escherichia coli 2011C-3911	99.82	0.0			
656408.ECPG_02507	ANE62009.1	Escherichia coli 2011C-3911	99.11	0.0			
656408.ECPG_02507	ANJ35016.1	Escherichia coli S51	100.00	0.0			
656408.ECPG_02507	ANK09557.1	Escherichia coli ER1821R	99.64	0.0			
656408.ECPG_02507	ANO29894.1	Escherichia coli EC590	99.82	0.0			
656408.ECPG_02507	ANO77677.1	Escherichia coli GB089	100.00	0.0			
656408.ECPG_02507	ANP07066.1	Escherichia coli 08-00022	99.28	0.0			

656408.ECPG_02507	ANP17771.1	Escherichia coli 09-00049	99.82	0.0
656408.ECPG_02507	ANP32306.1	Escherichia coli H1827/12	99.82	0.0
656408.ECPG_02507	ANQ00649.1	Escherichia coli 210205630	100.00	0.0
656408.ECPG_02507	ANW27520.1	Escherichia coli O177:H21	99.82	0.0
656408.ECPG_02507	AOM53099.1	Escherichia coli CFSAN004176	99.64	0.0
656408.ECPG_02507	AOO69629.1	Escherichia coli K-12 NEB 5-alpha	99.64	0.0
656408.ECPG_02507	AOR19630.1	Escherichia coli 210221272	99.64	0.0
656408.ECPG_02507	AOT33124.1	Escherichia coli FORC_031	99.64	0.0
656408.ECPG_02507	APA26551.1	Escherichia coli Y5	99.82	0.0
656408.ECPG_02507	APE53176.1	Escherichia coli MRSN352231	99.82	0.0
656408.ECPG_02507	APE58124.1	Escherichia coli MRSN346595	99.82	0.0
656408.ECPG_02507	APE63001.1	Escherichia coli MRSN346638	99.82	0.0
656408.ECPG_02507	APE67843.1	Escherichia coli MRSN346355	99.82	0.0
656408.ECPG_02507	APE80249.1	Escherichia coli FORC_029	99.82	0.0
656408.ECPG_02507	APE92312.1	Escherichia coli FORC_041	99.61	0.0
656408.ECPG_02507	APE92313.1	Escherichia coli FORC_041	98.99	0.0
656408.ECPG_02507	APG36669.1	Escherichia coli FMU073332	99.64	0.0
656408.ECPG_02507	APJ61492.1	Escherichia coli C2	99.82	0.0
656408.ECPG_02507	APJ69456.1	Escherichia coli C3	99.64	0.0
656408.ECPG_02507	APJ77690.1	Escherichia coli C5	99.64	0.0
656408.ECPG_02507	APJ81920.1	Escherichia coli C8	99.82	0.0
656408.ECPG_02507	APJ87378.1	Escherichia coli C9	99.82	0.0
656408.ECPG_02507	APJ92237.1	Escherichia coli C10	99.82	0.0
656408.ECPG_02507	APJ98662.1	Escherichia coli C11	99.82	0.0
656408.ECPG_02507	APJ99361.1	Escherichia coli D1	99.64	0.0
656408.ECPG_02507	APK06589.1	Escherichia coli D2	99.64	0.0
656408.ECPG_02507	APK10471.1	Escherichia coli D3	99.82	0.0
656408.ECPG_02507	APK15391.1	Escherichia coli D4	99.82	0.0
656408.ECPG_02507	APK27921.1	Escherichia coli D7	99.82	0.0
656408.ECPG_02507	APK38968.1	Escherichia coli D9	99.82	0.0
656408.ECPG_02507	APK50789.1	Escherichia coli H1	99.64	0.0
656408.ECPG_02507	APK53684.1	Escherichia coli H2	99.46	0.0
656408.ECPG_02507	APK63119.1	Escherichia coli H5	99.28	0.0
656408.ECPG_02507	APK67018.1	Escherichia coli H6	99.28	0.0
656408.ECPG_02507	APK69759.1	Escherichia coli H7	99.82	0.0
656408.ECPG_02507	APK77345.1	Escherichia coli H8	99.82	0.0
656408.ECPG_02507	APK79547.1	Escherichia coli H10	99.82	0.0
656408.ECPG_02507	APK86571.1	Escherichia coli H14	99.28	0.0
656408.ECPG_02507	APK88535.1	Escherichia coli H15	99.64	0.0
656408.ECPG_02507	APK92226.1	Escherichia coli M1	99.82	0.0
656408.ECPG_02507	APL01299.1	Escherichia coli M3	99.82	0.0
656408.ECPG_02507	APL02960.1	Escherichia coli M6	99.82	0.0
656408.ECPG_02507	APL10491.1	Escherichia coli M8	99.82	0.0
656408.ECPG_02507	APL12745.1	Escherichia coli M9	99.82	0.0
656408.ECPG_02507	APL20808.1	Escherichia coli M10	99.82	0.0
656408.ECPG_02507	APL23011.1	Escherichia coli M11	99.82	0.0
656408.ECPG_02507	APL28817.1	Escherichia coli M15	99.82	0.0
656408.ECPG_02507	APL37862.1	Escherichia coli M19	99.82	0.0
656408.ECPG_02507	APL43056.1	Escherichia coli S1	99.46	0.0
656408.ECPG_02507	APL47460.1	Escherichia coli S3	99.82	0.0
656408.ECPG_02507	APL53788.1	Escherichia coli S10	99.82	0.0
656408.ECPG_02507	APL55360.1	Escherichia coli S21	99.28	0.0
656408.ECPG_02507	APL59703.1	Escherichia coli S30	99.82	0.0
656408.ECPG_02507	APL66427.1	Escherichia coli S40	99.82	0.0
656408.ECPG_02507	APL70090.1	Escherichia coli S42	99.64	0.0
656408.ECPG_02507	APL77498.1	Escherichia coli S43	97.14	0.0
656408.ECPG_02507	APL81895.1	Escherichia coli S50	99.64	0.0
656408.ECPG_02507	APL86079.1	Escherichia coli C7	99.64	0.0
656408.ECPG_02507	APL91375.1	Escherichia coli S56	99.64	0.0
656408.ECPG_02507	APQ21787.1	Escherichia coli tolC-	99.64	0.0
656408.ECPG_02507	APW94787.1	Escherichia coli WCHEC050613	99.82	0.0
656408.ECPG_02507	AQP91219.1	Escherichia coli MGY	99.64	0.0
656408.ECPG_02507	AQT99101.1	Escherichia coli NCTC86	99.64	0.0
656408.ECPG_02507	AQV20769.1	Escherichia coli Ecol_224	100.00	0.0
656408.ECPG_02507	AQV37188.1	Escherichia coli Ecol_422	99.82	0.0
656408.ECPG_02507	AQV42663.1	Escherichia coli Ecol_517	99.82	0.0
656408.ECPG_02507	AQV79004.1	Escherichia coli Ecol_AZ147	99.46	0.0
656408.ECPG_02507	AQV90365.1	Escherichia coli Ecol_AZ155	99.82	0.0
656408.ECPG_02507	AQW72840.1	Escherichia coli M8	100.00	0.0
656408.ECPG_02507	AQZ30742.1	Escherichia coli USML2	99.64	0.0
656408.ECPG_02507	AQZ86645.1	Escherichia coli 13E0725	99.64	0.0
656408.ECPG_02507	ARA02901.1	Escherichia coli 13E0780	99.64	0.0
656408.ECPG_02507	ARA07993.1	Escherichia coli 13E0767	99.82	0.0
656408.ECPG_02507	ARA33529.1	Escherichia coli AR_0069	99.82	0.0
656408.ECPG_02507	ARD51102.1	Escherichia coli FAM21845	99.64	0.0
656408.ECPG_02507	ARD77010.1	Escherichia coli BLK9	99.64	0.0
656408.ECPG_02507	ARD80885.1	Escherichia coli BLK16	99.64	0.0
656408.ECPG_02507	ARE47878.1	Escherichia coli C	99.82	0.0
656408.ECPG_02507	ARH97073.1	Escherichia coli BLR(DE3)	99.82	0.0
656408.ECPG_02507	ARJ96202.1	Escherichia coli CFSAN051542	99.82	0.0
656408.ECPG_02507	ARM43182.1	Escherichia coli HST04	99.64	0.0
656408.ECPG_02507	ARM80105.1	Escherichia coli HB-Coli0	99.64	0.0
656408.ECPG_02507	ARQ26353.1	Escherichia coli CH611_eco	99.82	0.0
656408.ECPG_02507	ARR35427.1	Escherichia coli 5CRE51	99.82	0.0
656408.ECPG_02507	ARV36650.1	Escherichia coli KSC64	99.82	0.0
656408.ECPG_02507	ARV53085.1	Escherichia coli 1031	99.64	0.0
656408.ECPG_02507	ARW87711.1	Escherichia coli AR_0149	99.82	0.0
656408.ECPG_02507	ARW93063.1	Escherichia coli AR_0149	100.00	0.0
656408.ECPG_02507	ARX14408.1	Escherichia coli AR_0162	99.82	0.0
656408.ECPG_02507	ARX30667.1	Escherichia coli AR_0151	99.82	0.0
656408.ECPG_02507	ARZ81890.1	Escherichia coli AR_0114	99.82	0.0
656408.ECPG_02507	ARZ90106.1	Escherichia coli AR_0150	99.82	0.0
656408.ECPG_02507	ASB80482.1	Escherichia coli AR_0137	99.82	0.0
656408.ECPG_02507	ASF02022.1	Escherichia coli O104:H4 FDAARGOS_348	99.82	0.0
656408.ECPG_02507	ASG49571.1	Escherichia coli ABWA45	98.94	0.0
656408.ECPG_02507	ASI51133.1	Escherichia coli FORC_043	99.82	0.0
656408.ECPG_02507	ASJ43134.1	Escherichia coli PSUO103	99.46	0.0
656408.ECPG_02507	ASL31914.1	Escherichia coli E62	99.82	0.0
656408.ECPG_02507	ASO00343.1	Escherichia coli DS1	99.64	0.0
656408.ECPG_02507	ASO79606.1	Escherichia coli MEM	99.64	0.0
656408.ECPG_02507	ASO83058.1	Escherichia coli GE3	100.00	0.0
656408.ECPG_02507	ASO92563.1	Escherichia coli WAT	99.82	0.0
656408.ECPG_02507	ASQ66597.1	Escherichia coli NCCP15648	99.82	0.0
656408.ECPG_02507	ASZ41373.1	Escherichia coli 646	99.64	0.0
656408.ECPG_02507	ASZ45852.1	Escherichia coli 360/16	99.82	0.0
656408.ECPG_02507	ATB08117.1	Escherichia coli ETEC-2265	99.64	0.0
656408.ECPG_02507	ATB13280.1	Escherichia coli ETEC-2264	99.82	0.0
656408.ECPG_02507	ATB83348.1	Escherichia coli 1943	99.82	0.0
656408.ECPG_02507	ATB98198.1	Escherichia coli 1283	99.82	0.0
656408.ECPG_02507	ATC03046.1	Escherichia coli 127	99.64	0.0
656408.ECPG_02507	ATC07481.1	Escherichia coli 1223	99.82	0.0
656408.ECPG_02507	ATG05971.1	Escherichia coli FDAARGOS_401	99.82	0.0
656408.ECPG_02507	ATG09919.1	Escherichia coli FDAARGOS_403	99.82	0.0
656408.ECPG_02507	ATM12622.1	Escherichia coli FDAARGOS_434	99.82	0.0

656408.ECPG_02507	ATM28720.1	Escherichia coli FDAARGOS_433	99.82	0.0			
656408.ECPG_02507	ATO76038.1	Escherichia coli O91 str. RM7190	99.82	0.0			
656408.ECPG_02507	ATP22263.1	Escherichia coli UFU_EC98	99.82	0.0			
656408.ECPG_02507	ATU35244.1	Escherichia coli WG5	99.82	0.0			
656408.ECPG_02507	ATW98029.1	Escherichia coli CREC-629	99.82	0.0			
656408.ECPG_02507	ATX07930.1	Escherichia coli CREC-591	100.00	0.0			
656408.ECPG_02507	ATX20314.1	Escherichia coli CREC-532	99.82	0.0			
656408.ECPG_02507	ATX35860.1	Escherichia coli AMA1167	99.82	0.0			
656408.ECPG_02507	ATX46191.1	Escherichia coli AR_0011	99.82	0.0			
656408.ECPG_02507	ATX50646.1	Escherichia coli AR_0014	99.82	0.0			
656408.ECPG_02507	ATZ30474.1	Escherichia coli CV839-15	99.28	0.0			
656408.ECPG_02507	ATZ37582.1	Escherichia coli S17-13	99.64	0.0			
656408.ECPG_02507	AUA39233.1	Escherichia coli SC516	99.64	0.0			
656408.ECPG_02507	AUA46387.1	Escherichia coli S17-20	99.64	0.0			
656408.ECPG_02507	AUF75697.1	Escherichia coli O121:H19 16-9255	99.46	0.0			
656408.ECPG_02507	AUJ91066.1	Escherichia coli 14EC047	99.82	0.0			
656408.ECPG_02507	AUK01522.1	Escherichia coli 14EC029	99.82	0.0			
656408.ECPG_02507	AUK11801.1	Escherichia coli 14EC017	99.82	0.0			
656408.ECPG_02507	AUK17035.1	Escherichia coli 14EC007	99.82	0.0			
656408.ECPG_02507	AUL63841.1	Escherichia coli CRE1493	99.82	0.0			
656408.ECPG_02507	AUL70512.1	Escherichia coli CRE1540	99.82	0.0			
656408.ECPG_02507	AUL85442.1	Escherichia coli SMN197SH3	99.64	0.0			
656408.ECPG_02507	AUL89883.1	Escherichia coli SMN152SH1	99.64	0.0			
656408.ECPG_02507	AUM22805.1	Escherichia coli SMN013SH2	99.64	0.0			
656408.ECPG_02507	AUN47905.1	Escherichia coli SCEC020007	99.82	0.0			
656408.ECPG_02507	AUO42987.1	Escherichia coli Ec40	99.82	0.0			
656408.ECPG_02507	AUO57383.1	Escherichia coli ML35	99.64	0.0			
656408.ECPG_02507	AUP46755.1	Escherichia coli CV839-06	99.64	0.0			
656408.ECPG_02507	AUS38535.1	Escherichia coli SCEC020023	99.82	0.0			
656408.ECPG_02507	AUT09483.1	Escherichia coli DH5alpha	99.64	0.0			
656408.ECPG_02507	AUV19787.1	Escherichia coli ECONIH6	99.82	0.0			
656408.ECPG_02507	AUV29619.1	Escherichia coli ECONIH5	98.94	0.0			
656408.ECPG_02507	AUX02823.1	Escherichia coli FORC_042	99.82	0.0			
656408.ECPG_02507	AUY01290.1	Escherichia coli ECONIH4	98.94	0.0			
656408.ECPG_02507	AUY29529.1	Escherichia coli KBN10P04869	99.82	0.0			
656408.ECPG_02507	AVB45834.1	Escherichia coli WCHEC005237	99.82	0.0			
656408.ECPG_02507	AVD32756.1	Escherichia coli DTU-1	99.64	0.0			
656408.ECPG_02507	AVL11461.1	Escherichia coli EC11	99.82	0.0			
656408.ECPG_02507	AVL29087.1	Escherichia coli O104:H4 FDAARGOS_349	99.82	0.0			
656408.ECPG_02507	AVM02482.1	Escherichia coli AS19-RrmA-	99.82	0.0			
656408.ECPG_02507	AVN01481.1	Escherichia coli WCHEC025943	99.82	0.0			
656408.ECPG_02507	AVP29638.1	Escherichia coli RM14721	99.82	0.0			
656408.ECPG_02507	AVR73049.1	Escherichia coli 675SK2	99.82	0.0			
656408.ECPG_02507	AVS05486.1	Escherichia coli CFSAN064036	99.82	0.0			
656408.ECPG_02507	AVT63287.1	Escherichia coli 26561	99.82	0.0			
656408.ECPG_02507	AVT73481.1	Escherichia coli HKUOPY1	99.28	0.0			
656408.ECPG_02507	AVZ09629.1	Escherichia coli WCHEC005784	99.41	0.0			
656408.ECPG_02507	AVZ48906.1	Escherichia coli J53	99.64	0.0			
656408.ECPG_02507	AVZ53809.1	Escherichia coli ME8067	99.64	0.0			
656408.ECPG_02507	AWF10404.1	Escherichia coli AR437	99.82	0.0			
656408.ECPG_02507	AWF22756.1	Escherichia coli AR435	99.82	0.0			
656408.ECPG_02507	AWF28068.1	Escherichia coli AR434	99.82	0.0			
656408.ECPG_02507	AWI98948.1	Escherichia coli H9Ecoli	99.82	0.0			
656408.ECPG_02507	AWJ26661.1	Escherichia coli O121 str. RM8352	99.46	0.0			
656408.ECPG_02507	AWJ99602.1	Escherichia coli ECCRA-119	99.82	0.0			
656408.ECPG_02507	AWM69948.1	Escherichia coli VH1	99.64	0.0			
656408.ECPG_02507	AWO21530.1	Escherichia coli DA33133	99.82	0.0			
656408.ECPG_02507	AWS65750.1	Escherichia coli AR_0085	99.82	0.0			
656408.ECPG_02507	AWU90769.1	Escherichia coli HS30-1	100.00	0.0			
656408.ECPG_02507	AWV10789.1	Escherichia coli 51008369SK1	99.82	0.0			
656408.ECPG_02507	AWX38772.1	Escherichia coli WCHEC035148	99.82	0.0			
656408.ECPG_02507	AWY88767.1	Escherichia coli	99.82	0.0			
656408.ECPG_02507	AWZ68329.1	Escherichia coli AR_452	99.82	0.0			
656408.ECPG_02507	AXA08721.1	Escherichia coli RTdelA_B_UU3	99.64	0.0			
656408.ECPG_02507	AXE57637.1	Escherichia coli AMSHJX01	99.82	0.0			
656408.ECPG_02507	AXI35675.1	Escherichia coli FORC 064	99.64	0.0			
656408.ECPG_02507	AXL03900.1	Escherichia coli ST410	99.82	0.0			
656408.ECPG_02507	AXL18024.1	Escherichia coli C600	99.64	0.0			
656408.ECPG_02507	AXO06955.1	Escherichia coli cq9	99.64	0.0			
656408.ECPG_02507	AXP27026.1	Escherichia coli UK_Dog_Liverpool	99.82	0.0			
656408.ECPG_02507	BAA14886.1	Escherichia coli str. K-12 substr. W3110	99.64	0.0			
656408.ECPG_02507	BAG76885.1	Escherichia coli SE11	99.82	0.0			
656408.ECPG_02507	BAI30302.1	Escherichia coli O103:H2 str. 12009	99.46	0.0			
656408.ECPG_02507	BAL38373.1	Escherichia coli str. K-12 substr. MDS42	99.64	0.0			
656408.ECPG_02507	CAQ31814.2	Escherichia coli BL21(DE3)	99.82	0.0			
656408.ECPG_02507	CAQ98193.1	Escherichia coli IAI1	99.82	0.0			
656408.ECPG_02507	CAU97329.1	Escherichia coli 55989	99.82	0.0			
656408.ECPG_02507	CBJ00916.1	Escherichia coli ETEC H10407	99.64	0.0			
656408.ECPG_02507	CCQ28508.2	Escherichia coli HUSEC2011	99.82	0.0			
656408.ECPG_02507	CDJ71725.1	Escherichia coli str. K-12 substr. MC4100	99.64	0.0			
656408.ECPG_02507	CDY57626.1	Escherichia coli K-12 substr. HMS174	99.64	0.0			
656408.ECPG_02507	CDZ20139.1	Escherichia coli K-12 substr. RV308	99.64	0.0			
656408.ECPG_02507	CQR80868.1	Escherichia coli K-12	99.64	0.0			
656408.ECPG_02507	CUQ96390.1	Escherichia coli MC1061	99.64	0.0			
656408.ECPG_02507	SCA70990.1	Escherichia coli	99.82	0.0			
656408.ECPG_02507	SMB29439.1	Escherichia coli	99.82	0.0			
656408.ECPG_02507	SMB29440.1	Escherichia coli	99.82	0.0			
656408.ECPG_02507	SNW13755.1	Escherichia coli NCTC122	99.82	0.0			
656408.ECPG_02507	SQE44352.1	Escherichia coli NCTC9967	99.60	0.0			
656408.ECPG_02507	SQE53724.1	Escherichia coli NCTC11023	96.96	0.0			
656419.ECJG_00789	ADA73717.1	Shigella flexneri 2002017	97.29	0.0	181	14.29	3.13088e-05
656419.ECJG_00789	AHG08418.1	Escherichia coli O145:H28 str. RM13514	97.85	0.0			
656419.ECJG_00789	AHG14368.1	Escherichia coli O145:H28 str. RM13516	98.03	0.0			
656419.ECJG_00789	AHY64705.1	Escherichia coli O145:H28 str. RM12761	98.03	0.0			
656419.ECJG_00789	AHY70241.1	Escherichia coli O145:H28 str. RM12581	97.85	0.0			
656419.ECJG_00789	AIL35420.1	Shigella flexneri 2003036	97.29	0.0			
656419.ECJG_00789	AIL40344.1	Shigella flexneri Shi06HN006	97.29	0.0			
656419.ECJG_00789	AJA25782.1	Escherichia coli O157:H7 str. SS52	98.93	0.0			
656419.ECJG_00789	AKK53902.1	Shigella flexneri G1663	97.29	0.0			
656419.ECJG_00789	ALH90358.1	Escherichia coli O157:H7 WS4202	98.85	0.0			
656419.ECJG_00789	AMG78620.1	Escherichia coli O157:H7 JEONG-1266	98.85	0.0			
656419.ECJG_00789	AMW41343.1	Escherichia coli 28RC1	98.85	0.0			
656419.ECJG_00789	AMW51403.1	Escherichia coli SRCC 1675	98.85	0.0			
656419.ECJG_00789	ANE67060.1	Escherichia coli 2013C-4465	98.85	0.0			
656419.ECJG_00789	ANG68573.1	Escherichia coli O157:H7 FRIK2533	98.85	0.0			
656419.ECJG_00789	ANG74072.1	Escherichia coli O157:H7 FRIK2455	98.85	0.0			
656419.ECJG_00789	ANG79620.1	Escherichia coli O157:H7 FRIK2069	98.85	0.0			
656419.ECJG_00789	ANW39980.1	Escherichia coli O157:H7 FRIK944	98.85	0.0			
656419.ECJG_00789	AOD11513.1	Escherichia coli EDL933-1	98.85	0.0			
656419.ECJG_00789	AOV21776.1	Escherichia coli O157:H7 1130	98.85	0.0			
656419.ECJG_00789	AOV27319.1	Escherichia coli O157:H7 2149	98.85	0.0			
656419.ECJG_00789	AOV32674.1	Escherichia coli O157:H7 2159	98.85	0.0			
656419.ECJG_00789	AOV38022.1	Escherichia coli O157:H7 3384	98.85	0.0			
656419.ECJG_00789	AOV43260.1	Escherichia coli O157:H7 4276	98.85	0.0			

656419.ECJG_00789	AOV48778.1	Escherichia coli O157:H7 8368	98.85	0.0			
656419.ECJG_00789	AOV54206.1	Escherichia coli O157:H7 9234	98.85	0.0			
656419.ECJG_00789	AOX52692.1	Escherichia coli NADC 5570/86-24/6565	98.85	0.0			
656419.ECJG_00789	AOX58085.1	Escherichia coli NADC 5570/86-24/6564	98.85	0.0			
656419.ECJG_00789	APA42687.1	Escherichia coli PA20	98.85	0.0			
656419.ECJG_00789	APH98020.1	Escherichia coli 155	98.85	0.0			
656419.ECJG_00789	API03650.1	Escherichia coli 272	98.85	0.0			
656419.ECJG_00789	API09485.1	Escherichia coli 319	98.85	0.0			
656419.ECJG_00789	API14993.1	Escherichia coli 350	98.85	0.0			
656419.ECJG_00789	API20171.1	Escherichia coli 472	98.85	0.0			
656419.ECJG_00789	API26070.1	Escherichia coli 7784	98.85	0.0			
656419.ECJG_00789	API31530.1	Escherichia coli 9000	98.85	0.0			
656419.ECJG_00789	API37310.1	Escherichia coli 10671	98.85	0.0			
656419.ECJG_00789	APK57915.1	Escherichia coli H3	95.89	0.0			
656419.ECJG_00789	AQZ77746.1	Escherichia coli DSM 103246	99.11	0.0			
656419.ECJG_00789	ARA15751.1	Escherichia coli AR_0118	95.89	0.0			
656419.ECJG_00789	ARV31842.1	Escherichia coli 9	95.89	0.0			
656419.ECJG_00789	ARV50990.1	Escherichia coli 207	94.96	0.0			
656419.ECJG_00789	ARX56851.1	Escherichia coli AR_0128	95.89	0.0			
656419.ECJG_00789	ASE50280.1	Escherichia coli O157 FDAARGOS_293	98.85	0.0			
656419.ECJG_00789	ASL59332.1	Escherichia coli FORC_044	98.85	0.0			
656419.ECJG_00789	ASQ53003.1	Shigella flexneri 4c 1602	97.29	0.0			
656419.ECJG_00789	ASQ56830.1	Shigella flexneri 4c 0702	97.29	0.0			
656419.ECJG_00789	ASQ63642.1	Shigella flexneri 1a 0439	97.29	0.0			
656419.ECJG_00789	ASQ82301.1	Shigella flexneri 1a 0670	97.29	0.0			
656419.ECJG_00789	AST66394.1	Escherichia coli CDC#03-98	98.85	0.0			
656419.ECJG_00789	ATH67736.1	Shigella flexneri 1c Y394	97.02	0.0			
656419.ECJG_00789	AWJ05203.1	Escherichia coli H8Ecoli	95.89	0.0			
656419.ECJG_00789	AWJ48643.1	Escherichia coli O43 str. RM10042	95.89	0.0			
656419.ECJG_00789	AWN68744.1	Escherichia coli RM10466	95.89	0.0			
656419.ECJG_00789	AWN77593.1	Escherichia coli O145 str. RM9872	97.85	0.0			
656419.ECJG_00789	AXC56685.1	Shigella flexneri 2a 1508	97.35	0.0			
656419.ECJG_00789	AXF72144.1	Escherichia coli AMSCJX02	95.89	0.0			
656419.ECJG_00789	BAB35310.1	Escherichia coli O157:H7 str. Sakai	98.47	0.0			
656419.ECJG_00789	CDX06624.1	Shigella flexneri	97.29	0.0			
656419.ECJG_00789	NP_287879.1	Escherichia coli O157:H7 str. EDL933	98.75	0.0			
V1.UC21-4_GL0152588	AIE64133.1	Enterobacter cloacae ECNIH2	99.11	0.0	55	4.34	1.40184e-05
V1.UC21-4_GL0152588	AJB63082.1	Enterobacter hormaechei subsp. steigerwaltii 34977	98.94	0.0			
V1.UC21-4_GL0152588	AJB71207.1	Enterobacter hormaechei subsp. hormaechei 34983	96.28	0.0			
V1.UC21-4_GL0152588	AJB82057.1	Enterobacter hormaechei subsp. oharae 34399	96.64	0.0			
V1.UC21-4_GL0152588	AKK79395.1	Enterobacter cloacae CAV1311	99.11	0.0			
V1.UC21-4_GL0152588	AKK92438.1	Enterobacter cloacae CAV1411	99.11	0.0			
V1.UC21-4_GL0152588	AKK96411.1	Enterobacter cloacae CAV1668	99.11	0.0			
V1.UC21-4_GL0152588	AKL51780.1	Enterobacter cloacae CAV1669	99.11	0.0			
V1.UC21-4_GL0152588	AKZ84479.1	Enterobacter hormaechei subsp. steigerwaltii 34998	99.12	0.0			
V1.UC21-4_GL0152588	ALA02383.1	Enterobacter hormaechei subsp. oharae 34978	96.81	0.0			
V1.UC21-4_GL0152588	ALV83403.1	Enterobacter cloacae G6809	98.05	0.0			
V1.UC21-4_GL0152588	ANS17569.1	Enterobacter hormaechei CAV1176	99.11	0.0			
V1.UC21-4_GL0152588	AOP78264.1	Enterobacter hormaechei subsp. steigerwaltii DSM 16691	99.29	0.0			
V1.UC21-4_GL0152588	AOP83094.1	Enterobacter hormaechei subsp. oharae DSM 16687	98.41	0.0			
V1.UC21-4_GL0152588	AOP91594.1	Enterobacter hormaechei subsp. xiangfangensis LMG27195	96.99	0.0			
V1.UC21-4_GL0152588	APR41473.1	Enterobacter cloacae AR_0002	96.81	0.0			
V1.UC21-4_GL0152588	ARA29483.1	Enterobacter cloacae AR_0065	99.82	0.0			
V1.UC21-4_GL0152588	ARZ81273.1	Enterobacter cloacae AR_0163	98.05	0.0			
V1.UC21-4_GL0152588	ASA06867.1	Enterobacter cloacae AR_0053	96.61	0.0			
V1.UC21-4_GL0152588	ASB82762.1	Enterobacter cloacae AR_0136	96.81	0.0			
V1.UC21-4_GL0152588	ASP02050.1	Enterobacter cloacae MS7884A	99.47	0.0			
V1.UC21-4_GL0152588	ATW92927.1	Enterobacter sp. CRENT-193	96.64	0.0			
V1.UC21-4_GL0152588	AVE75308.1	Enterobacter cloacae AR_0060	98.41	0.0			
V1.UC21-4_GL0152588	AVF15456.1	Enterobacter cloacae AR_0072	98.41	0.0			
V1.UC21-4_GL0152588	AVO84011.1	Enterobacter cloacae AR_0093	96.64	0.0			
V1.UC21-4_GL0152588	AWF30692.1	Enterobacter hormaechei AR432	98.75	0.0			
V1.UC21-4_GL0152588	AWQ43752.1	Enterobacter cloacae 234	97.35	0.0			
V1.UC21-4_GL0152588	AWQ57964.1	Enterobacter cloacae 388	97.35	0.0			
V1.UC21-4_GL0152588	AWR68818.1	Enterobacter hormaechei subsp. xiangfangensis OSUKPC4_L	96.81	0.0			
V1.UC21-4_GL0152588	AWS77924.1	Enterobacter cloacae AR_0154	99.12	0.0			
V1.UC21-4_GL0152588	AWV76028.1	Enterobacter hormaechei subsp. xiangfangensis Pb204	99.47	0.0			
V1.UC21-4_GL0152588	AWX04181.1	Enterobacter cloacae 20710	96.99	0.0			
V1.UC21-4_GL0152588	AWZ97512.1	Enterobacter cloacae AR_038	99.11	0.0			
V1.UC21-4_GL0152588	AXL99615.1	Enterobacter hormaechei subsp. xiangfangensis OSUVMCKPC4-2	96.81	0.0			
V1.UC21-4_GL0152588	AXO40336.1	Enterobacter hormaechei S5	98.76	0.0			
V1.UC21-4_GL0152588	AXO47083.1	Enterobacter hormaechei S13	99.12	0.0			
V1.UC21-4_GL0152588	AXO51547.1	Enterobacter hormaechei S6	99.12	0.0			
V1.UC21-4_GL0152588	AXQ33045.1	Enterobacter hormaechei WCHEH020038	97.17	0.0			
V1.UC21-4_GL0152588	SMF92936.1	Enterobacter cloacae DG6	99.65	0.0			
DOM005_GL0051693	AIN22928.1	Enterobacter hormaechei subsp. hoffmannii ECNIH3	99.29	0.0	30	2.37	1.62598e-05
DOM005_GL0051693	AIN28266.1	Enterobacter hormaechei subsp. hoffmannii ECR091	99.29	0.0			
DOM005_GL0051693	AIX59123.1	Enterobacter cloacae ECNIH5	99.29	0.0			
DOM005_GL0051693	AOQ00148.1	Enterobacter hormaechei subsp. hoffmannii DSM 14563	99.65	0.0			
DOM005_GL0051693	ASB75288.1	Enterobacter cloacae AR_0050	98.94	0.0			
DOM005_GL0051693	AVJ80092.1	Enterobacter hormaechei subsp. hoffmannii AR_0365	99.64	0.0			
DOM005_GL0051693	AVZ14557.1	Enterobacter hormaechei SCEH020042	99.82	0.0			
T2D-108A_GL0194913	AFM60203.1	Enterobacter cloacae subsp. dissolvens SDM	98.57	0.0	5	0.39	1.70435e-05
1045856.EcWSU1_02474	AEW73909.1	Enterobacter cloacae EcWSU1	100.00	0.0	16	1.26	2.37877e-06
1045856.EcWSU1_02474	AHE70389.1	Enterobacter cloacae P101	98.93	0.0			
1045856.EcWSU1_02474	AKM87790.1	Enterobacter cloacae UW5	99.12	0.0			
1045856.EcWSU1_02474	AOT43270.1	Enterobacter ludwigii EN-119	99.47	0.0			
1045856.EcWSU1_02474	AVP02658.1	Enterobacter cloacae complex sp. FDA-CDC-AR_0132	99.47	0.0			
1045856.EcWSU1_02474	AWC86281.1	Enterobacter cloacae complex sp. FDA-CDC-AR_0164	99.65	0.0			
MH0179_GL0059381	AKL16517.1	Citrobacter freundii CAV1321	90.16	0.0	11	0.87	4.58739e-06
MH0179_GL0059381	AKL55249.1	Citrobacter freundii CAV1741	90.16	0.0			
MH0179_GL0059381	ANZ87250.1	Citrobacter freundii B38	90.71	0.0			
MH0179_GL0059381	AOI31289.1	Citrobacter freundii SL151	90.36	0.0			
MH0179_GL0059381	APR33611.1	Citrobacter freundii BD	91.09	0.0			
MH0179_GL0059381	ARC40160.1	Citrobacter braakii FDAARGOS_253	90.73	0.0			
MH0179_GL0059381	ASE41675.1	Citrobacter braakii FDAARGOS_290	91.27	0.0			
MH0179_GL0059381	ASG44264.1	Citrobacter freundii 705SK3	90.71	0.0			
MH0179_GL0059381	ASJ99159.1	Citrobacter freundii 18-1	90.71	0.0			
MH0179_GL0059381	ATX02905.1	Citrobacter freundii CRCB-101	91.09	0.0			
MH0179_GL0059381	AUT96960.1	Citrobacter freundii FDAARGOS_61	90.73	0.0			
MH0179_GL0059381	AUV26158.1	Citrobacter freundii complex sp. CFNIH3	91.27	0.0			
MH0179_GL0059381	AUZ69639.1	Citrobacter freundii complex sp. CFNIH4	90.36	0.0			
MH0179_GL0059381	AVQ89635.1	Citrobacter freundii B9-C2	90.36	0.0			
MH0179_GL0059381	AWS96803.1	Citrobacter sp. CRE-46 AR_0156	96.44	0.0			
MH0179_GL0059381	BBA25990.1	Enterobacter hormaechei TUM11043	90.87	0.0			
MH0277_GL0009193	AAQ45175.1	Enterococcus faecium	99.47	0.0	47	3.71	1.82128e-05
MH0277_GL0009193	AFC64909.1	Enterococcus faecium Aus0004	99.47	0.0			
MH0277_GL0009193	AFK60287.1	Enterococcus faecium DO	99.47	0.0			
MH0277_GL0009193	AGE31270.1	Enterococcus faecium NRRL B-2354	99.64	0.0			
MH0277_GL0009193	AGS76839.1	Enterococcus faecium Aus0085	99.29	0.0			
MH0277_GL0009193	AII40086.1	Enterococcus faecium T110	98.40	0.0			
MH0277_GL0009193	ALF49749.1	Enterococcus faecium 64/3	99.47	0.0			
MH0277_GL0009193	ALL09020.1	Enterococcus faecium UW7606x64/3 TC1	99.47	0.0			
MH0277_GL0009193	ALZ53339.1	Enterococcus faecium 6E6	99.29	0.0			

MH0277_GL0009193	AMP60108.1	Enterococcus faecium UW8175	100.00	0.0			
MH0277_GL0009193	AMQ98417.1	Enterococcus faecium ATCC 700221	99.47	0.0			
MH0277_GL0009193	ANB95031.1	Enterococcus faecium E39	99.47	0.0			
MH0277_GL0009193	AOM17049.1	Enterococcus faecium ISMMS_VRE_1	99.47	0.0			
MH0277_GL0009193	AOM17857.1	Enterococcus faecium ISMMS_VRE_2	99.47	0.0			
MH0277_GL0009193	AOM22256.1	Enterococcus faecium ISMMS_VRE_3	99.47	0.0			
MH0277_GL0009193	AOM26458.1	Enterococcus faecium ISMMS_VRE_4	99.47	0.0			
MH0277_GL0009193	AOM27204.1	Enterococcus faecium ISMMS_VRE_5	99.47	0.0			
MH0277_GL0009193	AOM32025.1	Enterococcus faecium ISMMS_VRE_7	99.47	0.0			
MH0277_GL0009193	AOM34008.1	Enterococcus faecium ISMMS_VRE_8	99.47	0.0			
MH0277_GL0009193	AOM38253.1	Enterococcus faecium ISMMS_VRE_10	99.47	0.0			
MH0277_GL0009193	AON59726.1	Enterococcus faecium ISMMS_VRE_11	99.47	0.0			
MH0277_GL0009193	AOT79967.1	Enterococcus faecium E745	99.47	0.0			
MH0277_GL0009193	APJ07632.1	Enterococcus faecium VRE001	99.47	0.0			
MH0277_GL0009193	APV55597.1	Enterococcus faecium ISMMS_VRE_12	99.47	0.0			
MH0277_GL0009193	APV58385.1	Enterococcus faecium ISMMS_VRE_9	99.47	0.0			
MH0277_GL0009193	AQT57736.1	Enterococcus faecium VREF	99.29	0.0			
MH0277_GL0009193	AQY29852.1	Enterococcus faecium isolate 2014-VREF-63	99.29	0.0			
MH0277_GL0009193	AQY31370.1	Enterococcus faecium isolate 2014-VREF-268	99.47	0.0			
MH0277_GL0009193	ATD79535.1	Enterococcus faecium K60-39	99.29	0.0			
MH0277_GL0009193	ATU01452.1	Enterococcus faecium E240	99.47	0.0			
MH0277_GL0009193	ATU04403.1	Enterococcus faecium E243	99.47	0.0			
MH0277_GL0009193	ATU30587.1	Enterococcus faecium 16-346	99.29	0.0			
MH0277_GL0009193	ATW37429.1	Enterococcus faecium E232	99.47	0.0			
MH0277_GL0009193	AUC71705.1	Enterococcus faecium LS170308	100.00	0.0			
MH0277_GL0009193	AUH48806.1	Enterococcus faecium SC4	99.29	0.0			
MH0277_GL0009193	AUI19932.1	Enterococcus faecium Efaecium_ER04120.3A	99.47	0.0			
MH0277_GL0009193	AUI22901.1	Enterococcus faecium Efaecium_ER04462.3A	99.47	0.0			
MH0277_GL0009193	AUI25875.1	Enterococcus faecium Efaecium_ER04484.3A	99.47	0.0			
MH0277_GL0009193	AUI28849.1	Enterococcus faecium Efaecium_ER04526.5A	99.47	0.0			
MH0277_GL0009193	AUI34755.1	Enterococcus faecium Efaecium_ER04526.3A	99.47	0.0			
MH0277_GL0009193	AUJ66970.1	Enterococcus faecium WEFA23	98.57	0.0			
MH0277_GL0009193	AVJ43734.1	Enterococcus faecium 13-009	99.47	0.0			
MH0277_GL0009193	AVL45788.1	Enterococcus faecium FDAARGOS_323	99.47	0.0			
MH0277_GL0009193	AWV59697.1	Enterococcus faecium CFSAN059070	99.29	0.0			
MH0277_GL0009193	AWV62719.1	Enterococcus faecium CFSAN059071	99.29	0.0			
MH0277_GL0009193	CUX99927.1	Enterococcus faecium	97.15	0.0			
716541.ECL_01780	ADF61338.1	Enterobacter cloacae subsp. cloacae ATCC 13047	100.00	0.0	35	2.76	3.2226e-06
716541.ECL_01780	AIV29987.1	Enterobacter cloacae GGT036	98.57	0.0			
716541.ECL_01780	AOE95760.1	Enterobacter cloacae isolate SBP-8	98.57	0.0			
716541.ECL_01780	ASQ18263.1	Enterobacter cloacae M12X01451	98.75	0.0			
716541.ECL_01780	AVL18976.1	Enterobacter cloacae Res2010EC27	98.04	0.0			
MH0425_GL0086260	AKL19332.1	Citrobacter freundii CAV1321	99.28	0.0	41	3.24	3.84945e-06
MH0425_GL0086260	AKL56772.1	Citrobacter freundii CAV1741	99.28	0.0			
MH0425_GL0086260	ASG46280.1	Citrobacter freundii 705SK3	99.64	0.0			
MH0425_GL0086260	AVQ90950.1	Citrobacter freundii B9-C2	99.09	0.0			
MH0126_GL0034640	WP_008399816	Clostridium sp. L2-50	98.35	0.0	376	29.68	0.000548039
MH0003_GL0082004	AEH55776.1	Streptococcus parasanguinis ATCC 15912	99.38	0.0	319	25.18	3.66166e-05
MH0003_GL0082004	AFJ26298.1	Streptococcus parasanguinis FW213	98.57	0.0			
MH0193_GL0146584	AAN24362.1	Bifidobacterium longum NCC2705	98.62	0.0	561	44.28	0.000161452
MH0193_GL0146584	AAO84039.1	Bifidobacterium longum SJ32	99.21	0.0			
MH0193_GL0146584	AEI98364.1	Bifidobacterium longum subsp. longum KACC 91563	99.02	0.0			
MH0193_GL0146584	AOL09592.1	Bifidobacterium longum 35624	99.02	0.0			
MH0193_GL0146584	AXF97810.1	Bifidobacterium longum subsp. longum BORI	99.02	0.0			
MH0193_GL0146584	BAF62433.1	Bifidobacterium longum subsp. longum JCM 1217	98.62	0.0			
MH0193_GL0146584	BAJ65776.1	Bifidobacterium longum subsp. longum JCM 1217	99.02	0.0			
MH0193_GL0146584	BAJ70243.1	Bifidobacterium longum subsp. infantis 157F	98.82	0.0			
O2.UC36-0_GL0013311	AIW43159.1	Bifidobacterium longum subsp. longum GT15	99.41	0.0	681	53.75	0.000415948
O2.UC36-0_GL0013311	ALO71760.1	Bifidobacterium longum subsp. longum NCIMB8809	99.61	0.0			
O2.UC36-0_GL0013311	ALO73625.1	Bifidobacterium longum subsp. longum CCUG30698	99.41	0.0			
O2.UC36-0_GL0013311	BAP82762.1	Bifidobacterium longum 105-A	99.21	0.0			
O2.UC36-0_GL0013311	CBK71263.1	Bifidobacterium longum subsp. longum F8	99.02	0.0			
DLF012_GL0007335	ABE94812.1	Bifidobacterium breve UCC2003	99.80	0.0	200	15.79	0.000254505
DLF012_GL0007335	AEF27678.1	Bifidobacterium breve ACS-071-V-Sch8b	98.43	0.0			
DLF012_GL0007335	AHJ14902.1	Bifidobacterium breve 12L	99.80	0.0			
DLF012_GL0007335	AHJ16673.1	Bifidobacterium breve JCM 7017	99.02	0.0			
DLF012_GL0007335	AHJ18438.1	Bifidobacterium breve JCM 7019	98.62	0.0			
DLF012_GL0007335	AHJ20344.1	Bifidobacterium breve NCFB 2258	98.82	0.0			
DLF012_GL0007335	AHJ22176.1	Bifidobacterium breve 689b	98.23	0.0			
DLF012_GL0007335	AHJ24012.1	Bifidobacterium breve S27	100.00	0.0			
DLF012_GL0007335	ALE14166.1	Bifidobacterium breve BR3	100.00	0.0			
DLF012_GL0007335	AOP00147.1	Bifidobacterium longum subsp. longum AH1206	100.00	0.0			
DLF012_GL0007335	AQM42247.1	Bifidobacterium breve LMC520	100.00	0.0			
DLF012_GL0007335	AUD68252.1	Bifidobacterium breve NRBB02	99.80	0.0			
DLF012_GL0007335	AUD70093.1	Bifidobacterium breve NRBB04	98.82	0.0			
DLF012_GL0007335	AUD80204.1	Bifidobacterium breve NRBB51	98.43	0.0			
DLF012_GL0007335	AUD84177.1	Bifidobacterium breve NRBB56	99.61	0.0			
DLF012_GL0007335	AUD86167.1	Bifidobacterium breve NRBB57	99.80	0.0			
DLF012_GL0007335	AUD90252.1	Bifidobacterium breve DRBB27	98.43	0.0			
DLF012_GL0007335	AUD92349.1	Bifidobacterium breve DRBB28	99.80	0.0			
DLF012_GL0007335	AUD96362.1	Bifidobacterium breve 082W48	100.00	0.0			
DLF012_GL0007335	AUD98250.1	Bifidobacterium breve 139W423	99.21	0.0			
DLF012_GL0007335	AUE00260.1	Bifidobacterium breve 180W83	99.02	0.0			
DLF012_GL0007335	AUE04356.1	Bifidobacterium breve CNCM I-4321	99.61	0.0			
DLF012_GL0007335	AUE06461.1	Bifidobacterium breve NRBB08	99.80	0.0			
DLF012_GL0007335	AUE08335.1	Bifidobacterium breve NRBB18	99.80	0.0			
DLF012_GL0007335	AUE10210.1	Bifidobacterium breve NRBB19	99.80	0.0			
DLF012_GL0007335	AUE12085.1	Bifidobacterium breve NRBB20	99.80	0.0			
DLF012_GL0007335	AUE13960.1	Bifidobacterium breve NRBB27	99.80	0.0			
DLF012_GL0007335	AUE15831.1	Bifidobacterium breve NRBB49	99.80	0.0			
DLF012_GL0007335	AUE17680.1	Bifidobacterium breve DRBB29	98.43	0.0			
DLF012_GL0007335	AUE19760.1	Bifidobacterium breve DRBB30	99.61	0.0			
DLF012_GL0007335	BAQ99126.1	Bifidobacterium breve DSM 20213 = JCM 1192	100.00	0.0			
O2.UC28-2_GL0074842	AAN01605.1	Bifidobacterium animalis subsp. lactis	99.60	0.0	98	7.73	7.27624e-05
O2.UC28-2_GL0074842	ACL28432.1	Bifidobacterium animalis subsp. lactis AD011	100.00	0.0			
O2.UC28-2_GL0074842	ACS45532.1	Bifidobacterium animalis subsp. lactis Bl-04	100.00	0.0			
O2.UC28-2_GL0074842	ACS47099.1	Bifidobacterium animalis subsp. lactis DSM 10140	100.00	0.0			
O2.UC28-2_GL0074842	ADC85157.1	Bifidobacterium animalis subsp. lactis BB-12	100.00	0.0			
O2.UC28-2_GL0074842	ADG32722.1	Bifidobacterium animalis subsp. lactis V9	100.00	0.0			
O2.UC28-2_GL0074842	AEK29583.1	Bifidobacterium animalis subsp. lactis CNCM I-2494	100.00	0.0			
O2.UC28-2_GL0074842	AEN75912.1	Bifidobacterium animalis subsp. lactis BLC1	100.00	0.0			
O2.UC28-2_GL0074842	AFI62366.1	Bifidobacterium animalis subsp. animalis ATCC 25527	99.60	0.0			
O2.UC28-2_GL0074842	AFJ15894.1	Bifidobacterium animalis subsp. lactis B420	100.00	0.0			
O2.UC28-2_GL0074842	AFJ17455.1	Bifidobacterium animalis subsp. lactis Bi-07	100.00	0.0			
O2.UC28-2_GL0074842	AGO51644.1	Bifidobacterium animalis subsp. lactis Bl12	100.00	0.0			
O2.UC28-2_GL0074842	AGW84362.1	Bifidobacterium animalis subsp. lactis ATCC 27673	93.86	0.0			
O2.UC28-2_GL0074842	AIA32185.1	Bifidobacterium animalis RH	100.00	0.0			
O2.UC28-2_GL0074842	AIZ15622.1	Bifidobacterium pseudolongum PV8-2	90.48	0.0			
O2.UC28-2_GL0074842	AJC75873.1	Bifidobacterium animalis subsp. lactis KLDS2.0603	100.00	0.0			
O2.UC28-2_GL0074842	AJD33249.1	Bifidobacterium animalis A6	100.00	0.0			
O2.UC28-2_GL0074842	AJD87912.1	Bifidobacterium animalis subsp. lactis BF052	100.00	0.0			
O2.UC28-2_GL0074842	ANU43431.1	Bifidobacterium animalis subsp. animalis YL2	99.41	0.0			
O2.UC28-2_GL0074842	ASL77072.1	Bifidobacterium animalis BL3	100.00	0.0			
O2.UC28-2_GL0074842	ASW24530.1	Bifidobacterium pseudolongum UMB-MBP-01	90.28	0.0			

O2.UC28-2_GL0074842	ATU20080.1	Bifidobacterium choerinum FMB-1	90.69	0.0			
O2.UC28-2_GL0074842	AXM93247.1	Bifidobacterium animalis subsp. lactis S7	100.00	0.0			
O2.UC28-2_GL0074842	AXQ17362.1	Bifidobacterium animalis subsp. lactis IDCC4301	100.00	0.0			
**MH0131_GL0101467**	**AAO33821.1**	**Bifidobacterium adolescentis DSM 20083**	**99.80**	**0.0**	**577**	**45.54**	**0.00029932**
MH0131_GL0101467	AII75493.1	Bifidobacterium adolescentis 22L	99.80	0.0			
MH0131_GL0101467	AJE05047.1	Bifidobacterium adolescentis BBMN23	99.80	0.0			
MH0131_GL0101467	AVT44549.1	Bifidobacterium adolescentis 1-11	99.40	0.0			
MH0131_GL0101467	BAF38859.1	Bifidobacterium adolescentis ATCC 15703	99.80	0.0			
MH0188_GL0103816	BAQ95689.1	Bifidobacterium angulatum DSM 20098 = JCM 7096	98.02	0.0	393	31.02	8.19933e-05
MH0230_GL0139113	BAR02741.1	Bifidobacterium pseudocatenulatum DSM 20438 = JCM 1200 = LMG 10505	99.60	0.0	437	34.49	0.00114584
MH0230_GL0139113	CBK71507.1	Bifidobacterium pseudocatenulatum D2CA	99.40	0.0			
MH0272_GL0199098	AIZ13729.1	Bifidobacterium kashiwanohense PV20-2	99.01	0.0	242	19.10	0.000100675
MH0272_GL0199098	BAQ28208.1	Bifidobacterium kashiwanohense JCM 15439 = DSM 21854	98.41	0.0			
MH0272_GL0199098	BAR01027.1	Bifidobacterium catenulatum DSM 16992 = JCM 1194 = LMG 11043	100.00	0.0			
V1.CD7-0-PN_GL0043748	ADB08794.1	Bifidobacterium dentium Bd1	99.80	0.0	142	11.21	4.67435e-05
V1.CD7-0-PN_GL0043748	BAQ26093.1	Bifidobacterium dentium JCM 1195 = DSM 20436	99.80	0.0			
518635.BIFANG_02319	AMK57427.1	Bifidobacterium angulatum GT102	100.00	0.0	84	6.63	1.62057e-05
V1.CD7-0-PN_GL0000583	AGH40383.1	Bifidobacterium thermophilum RBL67	96.62	0.0	2	0.16	4.33881e-05
511691.SmuNN2025_1133	AAN58596.1	Streptococcus mutans UA159	99.17	0.0	68	5.37	5.58176e-06
511691.SmuNN2025_1133	AFM81308.1	Streptococcus mutans GS-5	98.96	0.0			
511691.SmuNN2025_1133	AJD55242.1	Streptococcus mutans UA159-FR	99.37	0.0			
511691.SmuNN2025_1133	AMF85569.1	Streptococcus mutans NG8	99.17	0.0			
511691.SmuNN2025_1133	ARS62272.1	Streptococcus mutans KCOM 1054 (= ChDC YM3)	98.96	0.0			
511691.SmuNN2025_1133	AVM71638.1	Streptococcus mutans LAR01	99.17	0.0			
511691.SmuNN2025_1133	BAH88159.1	Streptococcus mutans NN2025	100.00	0.0			
511691.SmuNN2025_1133	BAL69465.1	Streptococcus mutans LJ23	99.40	0.0			
511691.SmuNN2025_1133	BAQ24527.1	Streptococcus troglodytae TKU 31	97.51	0.0			
**511691.SmuNN2025_1133**	**CAA30846.1**	**Streptococcus mutans INGBRITT / GS5**	**97.50**	**0.0**			
511691.SmuNN2025_1133	SQF48861.1	Streptococcus mutans NCTC10449	99.38	0.0			
765135172-stool1_revised_C812172_1_gene66964	CBL13462.1	Roseburia intestinalis XB6B4	97.58	0.0	583	46.01	0.000599983
MH0006_GL0203164	AWY97923.1	Blautia sp. N6H1-15 KCTC 15426	97.97	0.0	860	67.88	0.000446549
**MH0006_GL0203164**	**CCA61958.1**	**Ruminococcus gnavus E1**	**97.97**	**0.0**			
**NOM023_GL0014561**	**ACR74281.1**	**Eubacterium rectale ATCC 33656**	**98.38**	**0.0**	**913**	**72.06**	**0.000744108**
NOM023_GL0014561	CBK89380.1	[Eubacterium] rectale DSM 17629	98.98	0.0			
NOM023_GL0014561	CBK94328.1	[Eubacterium] rectale M104/1	99.19	0.0			
O2.CD1-0-PT_GL0059378	AGS04734.1	Streptococcus lutetiensis 033	99.38	0.0	58	4.58	4.76096e-05
O2.CD1-0-PT_GL0059378	ALT82017.1	Streptococcus infantarius ICDDRB-NRC-S5	99.17	0.0			
O2.CD1-0-PT_GL0059378	BAK29318.1	Streptococcus pasteurianus ATCC 43144	96.67	0.0			
O2.CD1-0-PT_GL0059378	CCF01511.1	Streptococcus macedonicus ACA-DC 198	99.58	0.0			
O2.CD1-0-PT_GL0059378	SQF41508.1	Streptococcus lutetiensis NCTC8738	99.17	0.0			
O2.CD1-0-PT_GL0059378	SQG55569.1	Streptococcus lutetiensis NCTC13774	100.00	0.0			
O2.CD1-0-PT_GL0059378	SQI07765.1	Streptococcus pasteurianus NCTC13784	96.67	0.0			
586220.HMPREF0555_1560	AAX33736.1	Leuconostoc mesenteroides NRRL B-1149	98.98	0.0	24	1.89	8.56007e-07
586220.HMPREF0555_1560	ABJ61506.1	Leuconostoc mesenteroides subsp. mesenteroides ATCC 8293	99.59	0.0			
586220.HMPREF0555_1560	ABW71903.1	Leuconostoc mesenteroides B-1355	99.39	0.0			
586220.HMPREF0555_1560	ADK62363.1	Leuconostoc mesenteroides MBFWRS-3(1)	98.78	0.0			
586220.HMPREF0555_1560	AET29793.1	Leuconostoc mesenteroides subsp. mesenteroides J18	99.39	0.0			
586220.HMPREF0555_1560	AHF18539.1	Leuconostoc mesenteroides KFRI-MG	99.59	0.0			
586220.HMPREF0555_1560	AKP36398.1	Leuconostoc mesenteroides subsp. dextranicum DSM 20484	99.19	0.0			
586220.HMPREF0555_1560	APE76152.1	Leuconostoc mesenteroides DRC1506	99.59	0.0			
586220.HMPREF0555_1560	API71478.1	Leuconostoc suionicum DSM 20241	98.78	0.0			
586220.HMPREF0555_1560	AQU48795.1	Leuconostoc mesenteroides subsp. mesenteroides DRC0211	99.39	0.0			
586220.HMPREF0555_1560	ARN62880.1	Leuconostoc mesenteroides subsp. mesenteroides BD1710	99.19	0.0			
586220.HMPREF0555_1560	ASR68574.1	Leuconostoc mesenteroides WiKim33	99.59	0.0			
586220.HMPREF0555_1560	AWV37281.1	Leuconostoc mesenteroides CBA7131	99.59	0.0			
**586220.HMPREF0555_1560**	**BAA14344.1**	**Leuconostoc mesenteroides ATCC 12291**	**90.70**	**0.0**			
586220.HMPREF0555_1560	BAX70034.1	Leuconostoc suionicum LT-38	98.78	0.0			
586220.HMPREF0555_1560	BAX72008.1	Leuconostoc mesenteroides LK-151	99.59	0.0			
MH0345_GL0184076	CED94009.1	Romboutsia ilealis CRIB	98.77	0.0	570	44.99	0.000157783
ED9A_GL0077731	ARI56983.1	Streptococcus salivarius ATCC 27945	98.97	0.0	239	18.86	5.9649e-05
ED9A_GL0077731	ARI59814.1	Streptococcus salivarius ATCC 25975	98.56	0.0			
ED9A_GL0077731	SQF75623.1	Streptococcus salivarius NCTC7366	98.56	0.0			
DLM012_GL0046437	ALZ53602.1	Enterococcus faecium 6E6	100.00	0.0	26	2.05	9.49445e-06
DLM012_GL0046437	AMP62490.1	Enterococcus faecium UW8175	99.38	0.0			
DLM012_GL0046437	AOM14759.1	Enterococcus faecium ISMMS_VRE_1	100.00	0.0			
DLM012_GL0046437	AQT58538.1	Enterococcus faecium VREF	99.79	0.0			
DLM012_GL0046437	AQY33507.1	Enterococcus faecium isolate 2014-VREF-268	100.00	0.0			
DLM012_GL0046437	AUH49069.1	Enterococcus faecium SC4	100.00	0.0			
DLM012_GL0046437	AVJ43906.1	Enterococcus faecium 13-009	100.00	0.0			
DLM012_GL0046437	AVJ46693.1	Enterococcus faecium 13-022	99.79	0.0			
DLM012_GL0046437	AWB15626.1	Enterococcus faecium HL1	100.00	0.0			
DLM012_GL0046437	AWV59852.1	Enterococcus faecium CFSAN059070	100.00	0.0			
DLM012_GL0046437	AWV62869.1	Enterococcus faecium CFSAN059071	100.00	0.0			
MH0400_GL0107993	ABQ83787.1	Lactobacillus reuteri DSM 20016	100.00	0.0	13	1.03	1.2103e-05
MH0400_GL0107993	AEI56886.1	Lactobacillus reuteri SD2112	100.00	0.0			
**MH0400_GL0107993**	**AGK37834.1**	**Lactobacillus reuteri LTH5448**	**99.59**	**0.0**			
MH0400_GL0107993	AGN98627.1	Lactobacillus reuteri I5007	100.00	0.0			
MH0400_GL0107993	AGR64687.1	Lactobacillus reuteri TD1	99.79	0.0			
MH0400_GL0107993	ANU51845.1	Lactobacillus reuteri I49	100.00	0.0			
MH0400_GL0107993	AWD63077.1	Lactobacillus reuteri WHH1689	99.38	0.0			
MH0400_GL0107993	BAG25968.1	Lactobacillus reuteri JCM 1112	100.00	0.0			
MH0400_GL0107993	CUU12009.1	Lactobacillus reuteri ATCC 53608	100.00	0.0			
O2.UC21-1_GL0008570	AJT49663.1	Lactobacillus mucosae LM1	99.79	0.0	93	7.34	0.000190068
V1.CD7-0-PN_GL0025100	AKP01754.1	Lactobacillus reuteri IRT	93.81	0.0	7	0.55	9.02923e-06
V1.CD7-0-PN_GL0025100	AMY14476.1	Lactobacillus reuteri ZLR003	93.81	0.0			
T2D-31A_GL0045848	AMS07529.1	Lactobacillus oris J-1	99.59	0.0	15	1.18	6.65498e-06
388919.SSA_1006	ABN44422.1	Streptococcus sanguinis SK36	100.00	0.0	127	10.02	9.49495e-06
388919.SSA_1006	SQF71464.1	Streptococcus sanguinis NCTC11086	98.96	0.0			
684738.LLKF_2259	ADA65829.1	Lactococcus lactis subsp. lactis KF147	100.00	0.0	6	0.47	6.43558e-07
684738.LLKF_2259	ATC62278.1	Lactococcus raffinolactis WiKim0068	97.30	0.0			
O2.CD1-0_GL0015459	AGY37964.1	Streptococcus sp. I-P16	98.12	0.0	263	20.76	1.95629e-05
O2.CD1-0_GL0015459	AGY39509.1	Streptococcus sp. I-G2	98.12	0.0			
O2.UC34-0_GL0078155	ADQ59566.1	Lactobacillus amylovorus GRL 1112	99.38	0.0	21	1.66	8.13506e-05
O2.UC34-0_GL0078155	ADZ07705.1	Lactobacillus acidophilus 30SC	100.00	0.0			
O2.UC34-0_GL0078155	AEA32401.1	Lactobacillus amylovorus GRL1118	100.00	0.0			
O2.UC34-0_GL0078155	AOG26270.1	Lactobacillus johnsonii BS15	99.79	0.0			
O2.UC34-0_GL0078155	AUX16275.1	Lactobacillus amylovorus JBD401	99.79	0.0			
V1.CD7-0-PN_GL0008089	APG67766.1	Lactobacillus delbrueckii subsp. lactis KCTC 3035	99.79	0.0	12	0.95	1.42703e-05
V1.CD7-0-PN_GL0008089	APG70160.1	Lactobacillus delbrueckii subsp. lactis KCCM 34717	99.58	0.0			
V1.CD7-0-PN_GL0008089	APG70679.1	Lactobacillus delbrueckii subsp. delbrueckii KCTC 13731	99.58	0.0			
V1.CD7-0-PN_GL0008089	APG72592.1	Lactobacillus delbrueckii subsp. jakobsenii ZN7a-9 = DSM 26046	99.79	0.0			
V1.CD7-0-PN_GL0008089	APG73940.1	Lactobacillus delbrueckii subsp. sunkii JCM 17838	99.79	0.0			
V1.CD7-0-PN_GL0008089	ARR37661.1	Lactobacillus delbrueckii subsp. delbrueckii TUA4408L	99.58	0.0			
V1.CD7-0-PN_GL0008089	ASW11009.1	Lactobacillus delbrueckii subsp. lactis DSM 20072	99.38	0.0			
DLM012_GL0036753	CBL50849.1	Lactobacillus crispatus ST1	99.38	0.0	17	1.34	2.11229e-05
T2D-143A_GL0001632	AAS08245.1	Lactobacillus johnsonii NCC 533	98.33	0.0	10	0.79	3.60764e-05
T2D-143A_GL0001632	AEB92607.1	Lactobacillus johnsonii DPC 6026	97.29	0.0			
T2D-143A_GL0001632	AOG26671.1	Lactobacillus johnsonii BS15	97.29	0.0			
T2D-143A_GL0001632	ARW75563.1	Lactobacillus johnsonii UMNLJ21	99.79	0.0			
T2D-143A_GL0001632	ARW76477.1	Lactobacillus johnsonii UMNLJ22	99.79	0.0			

T2D-143A_GL0001632	AXQ19806.1	Lactobacillus johnsonii IDCC9203	99.79	0.0			
1069533.Sinf_0203	AEZ61571.1	Streptococcus infantarius subsp. infantarius CJ18	100.00	0.0	28	2.21	7.68987e-06
1069533.Sinf_0203	ALT81551.1	Streptococcus gallolyticus ICDDRB-NRC-S1	93.33	0.0			
1069533.Sinf_0203	AQP41203.1	Streptococcus gallolyticus subsp. gallolyticus DSM 16831	94.17	0.0			
1069533.Sinf_0203	BAK27136.1	Streptococcus gallolyticus subsp. gallolyticus ATCC 43143	94.17	0.0			
1069533.Sinf_0203	CBI12687.1	Streptococcus gallolyticus UCN34	94.17	0.0			
1069533.Sinf_0203	CBZ47385.1	Streptococcus gallolyticus subsp. gallolyticus ATCC BAA-2069	94.17	0.0			
1069533.Sinf_0203	SQG78484.1	Streptococcus gallolyticus NCTC13773	94.17	0.0			
272621.LBA0507	AAO21861.1	Lactobacillus acidophilus NCFM	100.00	0.0	13	1.03	2.64401e-06
272621.LBA0507	AGK93717.1	Lactobacillus acidophilus La-14	100.00	0.0			
272621.LBA0507	AJP45959.1	Lactobacillus acidophilus FSI4	100.00	0.0			
272621.LBA0507	ASN46425.1	Lactobacillus acidophilus ATCC 53544	100.00	0.0			
272621.LBA0507	ASX14497.1	Lactobacillus acidophilus LA1	100.00	0.0			
272621.LBA0507	AVW86344.1	Lactobacillus acidophilus DSM 20079	100.00	0.0			
**272621.LBA1437**	**AAO21868.1**	**Lactobacillus acidophilus NCFM**	**100.00**	**0.0**	**14**	**1.10**	**2.98329e-06**
272621.LBA1437	AGK94598.1	Lactobacillus acidophilus La-14	100.00	0.0			
272621.LBA1437	AJP46768.1	Lactobacillus acidophilus FSI4	100.00	0.0			
272621.LBA1437	ASN47283.1	Lactobacillus acidophilus ATCC 53544	100.00	0.0			
272621.LBA1437	ASX15322.1	Lactobacillus acidophilus LA1	100.00	0.0			
272621.LBA1437	AVW87179.1	Lactobacillus acidophilus DSM 20079	100.00	0.0			
748671.LCRIS_00503	CBL49950.1	Lactobacillus crispatus ST1	100.00	0.0	5	0.39	2.70444e-07
525326.HMPREF0514_11643	ASY54590.1	Lactobacillus gasseri DSM 14869	96.62	0.0	43	3.39	1.09371e-05
864570.HMPREF9189_0227	BAX31497.1	Streptococcus pneumoniae KK0381	94.66	0.0	155	12.23	1.04684e-05
864570.HMPREF9189_0227	BBA09080.1	Streptococcus oralis subsp. tigurinus osk_001	98.54	0.0			
864570.HMPREF9189_0227	CBZ00101.1	Streptococcus oralis Uo5	97.92	0.0			
SZEY-40A_GL0011887	AHW94064.1	Enterobacter asburiae L1	93.69	0.0	54	4.26	1.43639e-05
SZEY-40A_GL0011887	AKK99801.1	Enterobacter asburiae CAV1043	91.89	0.0			
SZEY-40A_GL0011887	AKZ73706.1	Enterobacter cloacae complex sp. 35734	98.42	0.0			
SZEY-40A_GL0011887	ALL17884.1	Enterobacter sp. E20	93.02	0.0			
SZEY-40A_GL0011887	AMX04954.1	Enterobacter asburiae ENIPBJ-CG1	91.67	0.0			
SZEY-40A_GL0011887	AMY66143.1	Enterobacter cloacae colR/S	91.89	0.0			
SZEY-40A_GL0011887	AOL12055.1	Enterobacter sp. HK169	93.47	0.0			
SZEY-40A_GL0011887	AOP95838.1	Enterobacter roggenkampii DSM 16690	99.10	0.0			
SZEY-40A_GL0011887	AQT91398.1	Enterobacter cloacae R11	99.09	0.0			
SZEY-40A_GL0011887	ASD59450.1	Enterobacter cloacae complex sp. ECNIH7	92.34	0.0			
SZEY-40A_GL0011887	ASG38242.1	Enterobacter cloacae 704SK10	99.32	0.0			
SZEY-40A_GL0011887	AUM03935.1	Enterobacter sp. Crenshaw	92.57	0.0			
SZEY-40A_GL0011887	AVG33789.1	Enterobacter cloacae complex sp. FDAARGOS_77	92.12	0.0			
SZEY-40A_GL0011887	AVH19812.1	Lelliottia nimipressuralis SGAir0187	95.27	0.0			
760570.HMPREF0833_10343	ABP92721.1	Streptococcus suis 98HAH33	90.48	0.0	85	6.71	4.42905e-06
760570.HMPREF0833_10343	ADE31842.1	Streptococcus suis GZ1	90.48	0.0			
760570.HMPREF0833_10343	ADV70581.1	Streptococcus suis JS14	90.48	0.0			
760570.HMPREF0833_10343	AEB81819.1	Streptococcus suis ST3	90.48	0.0			
760570.HMPREF0833_10343	AER15678.1	Streptococcus suis SS12	90.48	0.0			
760570.HMPREF0833_10343	AER17727.1	Streptococcus suis D9	90.02	0.0			
760570.HMPREF0833_10343	AER19785.1	Streptococcus suis D12	90.25	0.0			
760570.HMPREF0833_10343	AER44706.1	Streptococcus suis A7	90.48	0.0			
760570.HMPREF0833_10343	AFR00815.1	Streptococcus suis S735	90.48	0.0			
760570.HMPREF0833_10343	AGG64897.1	Streptococcus suis SC070731	90.48	0.0			
760570.HMPREF0833_10343	AGW87747.1	Streptococcus suis YB51	90.48	0.0			
760570.HMPREF0833_10343	AGZ23643.1	Streptococcus suis T15	90.48	0.0			
760570.HMPREF0833_10343	AHF59270.1	Streptococcus suis 05HAS68	90.25	0.0			
760570.HMPREF0833_10343	AIG43977.1	Streptococcus suis 6407	90.48	0.0			
760570.HMPREF0833_10343	AKG40804.1	Streptococcus suis ZY05719	90.48	0.0			
760570.HMPREF0833_10343	ALA28420.1	Streptococcus suis NSUI002	90.25	0.0			
760570.HMPREF0833_10343	AML46937.1	Streptococcus suis NSUI060	90.02	0.0			
760570.HMPREF0833_10343	AMU80194.1	Streptococcus suis 90-1330	90.25	0.0			
760570.HMPREF0833_10343	AND00266.1	Streptococcus suis DN13	90.02	0.0			
760570.HMPREF0833_10343	AOM74985.1	Streptococcus suis GZ0565	90.02	0.0			
760570.HMPREF0833_10343	ARL70362.1	Streptococcus suis SC19	90.48	0.0			
760570.HMPREF0833_10343	ARX90789.1	Streptococcus suis LSM102	90.48	0.0			
760570.HMPREF0833_10343	ATZ03119.1	Streptococcus suis CZ130302	90.48	0.0			
760570.HMPREF0833_10343	AUA19441.1	Streptococcus suis AH681	90.02	0.0			
760570.HMPREF0833_10343	AUW22360.1	Streptococcus suis SS2-1	90.48	0.0			
760570.HMPREF0833_10343	AUW24394.1	Streptococcus suis CS100322	90.48	0.0			
760570.HMPREF0833_10343	AUW25702.1	Streptococcus suis HA0609	90.25	0.0			
760570.HMPREF0833_10343	AWL26309.1	Streptococcus suis HN105	90.02	0.0			
760570.HMPREF0833_10343	AWX95846.1	Streptococcus suis 0061	90.25	0.0			
760570.HMPREF0833_10343	AWX97841.1	Streptococcus suis 1081	90.25	0.0			
760570.HMPREF0833_10343	AXI65214.1	Streptococcus suis SH1510	90.25	0.0			
760570.HMPREF0833_10343	AXI67872.1	Streptococcus suis JSHA1003	90.48	0.0			
760570.HMPREF0833_10343	CAR46849.1	Streptococcus suis P1/7	90.48	0.0			
760570.HMPREF0833_10343	CAZ52175.1	Streptococcus suis SC84	90.48	0.0			
760570.HMPREF0833_10343	CAZ56303.1	Streptococcus suis BM407	90.48	0.0			
760570.HMPREF0833_10343	SQH00975.1	Streptococcus suis NCTC10234	90.48	0.0			
T2D-132A_GL0014546	AFP70303.1	Enterobacter cloacae subsp. cloacae ENHKU01	99.52	0.0	40	3.16	5.49191e-06
T2D-132A_GL0014546	AIX54703.1	Enterobacter cloacae ECNIH4	99.52	0.0			
T2D-132A_GL0014546	AMJ70495.1	Enterobacter cloacae isolate MBRL1077	95.65	0.0			
T2D-132A_GL0014546	AMZ78538.1	Enterobacter sp. ODB01	100.00	0.0			
T2D-132A_GL0014546	AOP87083.1	Enterobacter kobei DSM 13645	99.28	0.0			
MH0277_GL0021133	AJH00907.1	Clostridium beijerinckii NCIMB 14988	90.49	0.0	17	1.34	9.77707e-06
MH0277_GL0021133	ALP91351.1	Clostridium butyricum KNU-L09	100.00	0.0			
MH0277_GL0021133	ALS17218.1	Clostridium butyricum JKY6D1	97.69	0.0			
MH0277_GL0021133	ALS17846.1	Clostridium butyricum JKY6D1	100.00	0.0			
MH0277_GL0021133	ANF14972.1	Clostridium butyricum TOA	100.00	0.0			
MH0277_GL0021133	AOR94981.1	Clostridium butyricum TK520	100.00	0.0			
MH0277_GL0021133	APF22777.1	Clostridium butyricum CDC_51208	97.17	0.0			
656444.ECNG_03270	ALL90725.1	Escherichia coli 2009C-3133	97.89	0.0	79	6.24	4.83227e-05
656444.ECNG_03270	AOM62674.1	Escherichia coli CFSAN004177	95.26	0.0			
656444.ECNG_03270	APK44108.1	Escherichia coli D10	97.89	0.0			
656444.ECNG_03270	AQU97032.1	Escherichia coli MDR_56	98.16	0.0			
656444.ECNG_03270	AQV31938.1	Escherichia coli Ecol_316	99.21	0.0			
656444.ECNG_03270	ARR60567.1	Escherichia coli Z1002	99.21	0.0			
656444.ECNG_03270	ART16423.1	Escherichia coli 95JB1	95.25	0.0			
656444.ECNG_03270	ASJ33782.1	Escherichia coli PSUO78	97.89	0.0			
656444.ECNG_03270	ATM80274.1	Escherichia coli FDAARGOS_448	99.21	0.0			
656444.ECNG_03270	ATX43196.1	Escherichia coli AR_0006	99.21	0.0			
656444.ECNG_03270	ATX59558.1	Escherichia coli AR_0015	99.21	0.0			
656444.ECNG_03270	AUK06812.1	Escherichia coli 14EC020	97.89	0.0			
656444.ECNG_03270	AUK22179.1	Escherichia coli 14EC001	95.26	0.0			
656444.ECNG_03270	AUO36747.1	Escherichia coli YDC107	99.21	0.0			
656444.ECNG_03270	AVJ69998.1	Escherichia coli AR_0372	99.21	0.0			
656444.ECNG_03270	AVN09485.1	Escherichia coli AR_0374	95.71	0.0			
656444.ECNG_03270	AVS48217.1	Escherichia coli CFSAN018748	99.21	0.0			
656444.ECNG_03270	AWA17216.1	Escherichia coli WCHEC4533	99.21	0.0			
656444.ECNG_03270	AWF16841.1	Escherichia coli AR436	95.71	0.0			
656444.ECNG_03270	AWH71032.1	Escherichia coli 104	97.89	0.0			
656444.ECNG_03270	AWO33046.1	Escherichia coli DA33137	99.21	0.0			
656444.ECNG_03270	AXE68130.1	Escherichia coli KL53	95.85	0.0			
656444.ECNG_03270	AXF91980.1	Escherichia coli APEC O2-211	98.16	0.0			
656444.ECNG_03270	AXN83872.1	Escherichia coli WCHEC96200	99.21	0.0			
656444.ECNG_03270	BAX10722.1	Escherichia coli 20Ec-P-124	97.89	0.0			
656444.ECNG_03270	BBF47284.1	Escherichia coli E2855	95.25	0.0			

656444.ECNG_03270	BBF53075.1	Escherichia coli E2863	95.25	0.0			
656444.ECNG_03270	BBF59028.1	Escherichia coli E2865	95.25	0.0			
353496.LBU_0042	ABJ57747.1	Lactobacillus delbrueckii subsp. bulgaricus ATCC BAA-365	98.02	0.0	39	3.08	4.88042e-06
353496.LBU_0042	ADY84227.1	Lactobacillus delbrueckii subsp. bulgaricus 2038	100.00	0.0			
353496.LBU_0042	ALT46490.1	Lactobacillus delbrueckii subsp. bulgaricus MN-BM-F01	98.68	0.0			
353496.LBU_0042	AXI14199.1	Lactobacillus delbrueckii subsp. bulgaricus L99	99.66	0.0			
NLM027_GL0036283	AAK75965.1	Streptococcus pneumoniae TIGR4	96.76	0.0	115	9.08	1.10996e-05
NLM027_GL0036283	AAL00512.1	Streptococcus pneumoniae R6	96.76	0.0			
NLM027_GL0036283	AAT15575.1	Streptococcus pneumoniae	95.87	0.0			
NLM027_GL0036283	ABJ53943.1	Streptococcus pneumoniae D39	96.76	0.0			
NLM027_GL0036283	ACA36341.1	Streptococcus pneumoniae Hungary19A-6	97.05	0.0			
NLM027_GL0036283	ACB91119.1	Streptococcus pneumoniae CGSP14	96.76	0.0			
NLM027_GL0036283	ACO17210.1	Streptococcus pneumoniae 70585	96.76	0.0			
NLM027_GL0036283	ACO18566.1	Streptococcus pneumoniae JJA	96.76	0.0			
NLM027_GL0036283	ACO20167.1	Streptococcus pneumoniae P1031	96.76	0.0			
NLM027_GL0036283	ACO23943.1	Streptococcus pneumoniae Taiwan19F-14	97.05	0.0			
NLM027_GL0036283	ADI70351.1	Streptococcus pneumoniae TCH8431/19A	96.76	0.0			
NLM027_GL0036283	ADM85475.1	Streptococcus pneumoniae AP200	96.76	0.0			
NLM027_GL0036283	ADM92021.1	Streptococcus pneumoniae 670-6B	95.87	0.0			
NLM027_GL0036283	AFC95446.1	Streptococcus pneumoniae ST556	97.05	0.0			
NLM027_GL0036283	AFS43882.1	Streptococcus pneumoniae gamPNI0373	96.76	0.0			
NLM027_GL0036283	AGZ48385.1	Streptococcus pneumoniae A026	96.76	0.0			
NLM027_GL0036283	AHZ47204.1	Streptococcus sp. VT 162	98.82	0.0			
NLM027_GL0036283	AJD72856.1	Streptococcus pneumoniae NT_110_58	96.46	0.0			
NLM027_GL0036283	ALD68640.1	Streptococcus mitis KCOM 1350 (= ChDC B183)	96.76	0.0			
NLM027_GL0036283	ANO37675.1	Streptococcus pneumoniae D219	96.76	0.0			
NLM027_GL0036283	ANR74643.1	Streptococcus sp. oral taxon 064 W10853	97.94	0.0			
NLM027_GL0036283	AOG56676.1	Streptococcus pneumoniae D122	96.76	0.0			
NLM027_GL0036283	AOG58855.1	Streptococcus pneumoniae D141	96.76	0.0			
NLM027_GL0036283	APJ31186.1	Streptococcus pneumoniae SP49	97.05	0.0			
NLM027_GL0036283	APJ33195.1	Streptococcus pneumoniae SP61	96.76	0.0			
NLM027_GL0036283	APJ35220.1	Streptococcus pneumoniae SP64	96.76	0.0			
NLM027_GL0036283	ARD35477.1	Streptococcus pneumoniae Hu17	97.05	0.0			
NLM027_GL0036283	ARD37669.1	Streptococcus pneumoniae Hu15	97.05	0.0			
NLM027_GL0036283	ATF56164.1	Streptococcus oralis FDAARGOS_367	98.23	0.0			
NLM027_GL0036283	AUB33032.1	Streptococcus pneumoniae 19F	96.76	0.0			
NLM027_GL0036283	AUC46126.1	Streptococcus pneumoniae 11A	96.46	0.0			
NLM027_GL0036283	AUF85585.1	Streptococcus pneumoniae Xen35	96.76	0.0			
NLM027_GL0036283	AVD75788.1	Streptococcus pneumoniae 335	96.76	0.0			
NLM027_GL0036283	AVN86743.1	Streptococcus pneumoniae D39V	96.76	0.0			
NLM027_GL0036283	AVV92029.1	Streptococcus pneumoniae CP2215	97.05	0.0			
NLM027_GL0036283	AXJ89066.1	Streptococcus pneumoniae SPN XDR SMC1710-32	96.46	0.0			
NLM027_GL0036283	AXK92092.1	Streptococcus pneumoniae EF3030	97.05	0.0			
NLM027_GL0036283	BAW85350.1	Streptococcus pneumoniae KK0981	96.46	0.0			
NLM027_GL0036283	BAX33769.1	Streptococcus pneumoniae KK1157	96.46	0.0			
NLM027_GL0036283	BBA59836.1	Streptococcus pneumoniae MDRSPN001	97.35	0.0			
NLM027_GL0036283	CAR69671.1	Streptococcus pneumoniae ATCC 700669	96.76	0.0			
NLM027_GL0036283	CBW33316.1	Streptococcus pneumoniae OXC141	97.05	0.0			
NLM027_GL0036283	CBW35347.1	Streptococcus pneumoniae INV200	96.76	0.0			
NLM027_GL0036283	CBW37314.1	Streptococcus pneumoniae INV104	96.76	0.0			
NLM027_GL0036283	CCM07647.1	Streptococcus pneumoniae SPNA45	96.76	0.0			
NLM027_GL0036283	CCP31393.1	Streptococcus pneumoniae SPN994038 SNP994038	97.05	0.0			
NLM027_GL0036283	CCP33378.1	Streptococcus pneumoniae SPN034183 SNP034183	97.05	0.0			
NLM027_GL0036283	CCP35352.1	Streptococcus pneumoniae SPN994039 SNP994039	97.05	0.0			
NLM027_GL0036283	COT14529.1	Streptococcus pneumoniae NCTC7465	96.76	0.0			
NLM027_GL0036283	CRI62411.1	Streptococcus pneumoniae A66	97.05	0.0			
NLM027_GL0036283	SQG02735.1	Streptococcus pneumoniae NCTC7466	96.76	0.0			
NLM027_GL0036283	SQG34950.1	Streptococcus pneumoniae NCTC13276	96.76	0.0			
NLM027_GL0036283	SQG99370.1	Streptococcus pneumoniae NCTC11902	97.05	0.0			
HT8A_GL0006477	ACJ51257.1	Bifidobacterium longum subsp. infantis ATCC 15697 = JCM 1222 = DSM 20088	98.94	0.0	149	11.76	0.000142179
HT8A_GL0006477	ADG99593.1	Bifidobacterium longum subsp. longum JDM301	99.29	0.0			
HT8A_GL0006477	AIF89702.1	Bifidobacterium longum BXY01	99.29	0.0			
HT8A_GL0006477	AJT55532.1	Bifidobacterium longum	97.17	0.0			
HT8A_GL0006477	ALE08855.1	Bifidobacterium longum subsp. infantis BT1	98.94	0.0			
HT8A_GL0006477	AUD82175.1	Bifidobacterium breve NRBB52	98.94	0.0			
HT8A_GL0006477	AUD88275.1	Bifidobacterium breve DRBB26	98.94	0.0			
HT8A_GL0006477	AUD94439.1	Bifidobacterium breve 017W439	98.59	0.0			
HT8A_GL0006477	AUE02155.1	Bifidobacterium breve 215W447a	98.59	0.0			
HT8A_GL0006477	BAJ67724.1	Bifidobacterium longum subsp. infantis ATCC 15697 = JCM 1222 = DSM 20088	98.94	0.0			
HT8A_GL0006477	SDO29056.1	Bifidobacterium longum Su859	98.59	0.0			
**MH0028_GL0007151**	**ASM68470.1**	**Blautia hansenii DSM 20583**	**97.79**	**0.0**	**645**	**50.91**	**0.000278924**
MH0432_GL0161201	ACD98776.1	Bifidobacterium longum DJO10A	99.60	0.0	382	30.15	0.000133166
MH0432_GL0161201	ALE35293.1	Bifidobacterium longum BG7	99.60	0.0			

Figure S1: *Rg*SPP purification. A. *Rg*SPP was first purified on Ni-NTA column and eluted with 125 mM imidazole in MOPS buffer pH 7.4. B. *Rg*SPP was then purified using SEC S200 26/60 column using 50 mM HEPES pH 7.0 with NaCl 150 mM.

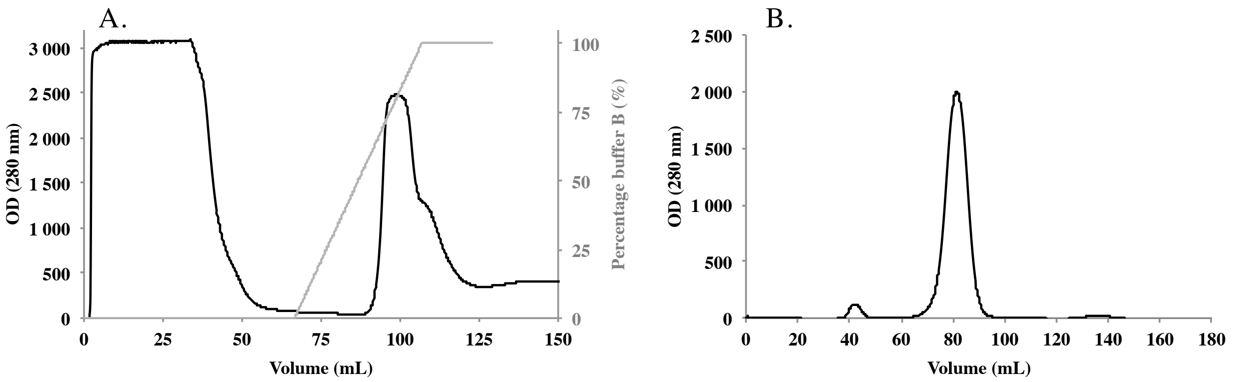

Figure S2: SDS-PAGE of the purified *Rg*SPP.

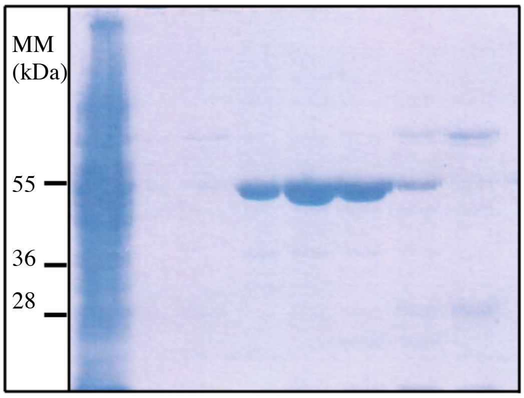

Figure S3: G1P production analysis. Sucrose and S6^{F}P were used as substrates in (A) and (B), respectively. The grey lines (full circle) correspond to NADPH released from the G1P production by *Rg*SPP, whereas dark lines (full square) correspond to the reaction with the SP from *Leuconostoc mesenteroides* used as positive control.

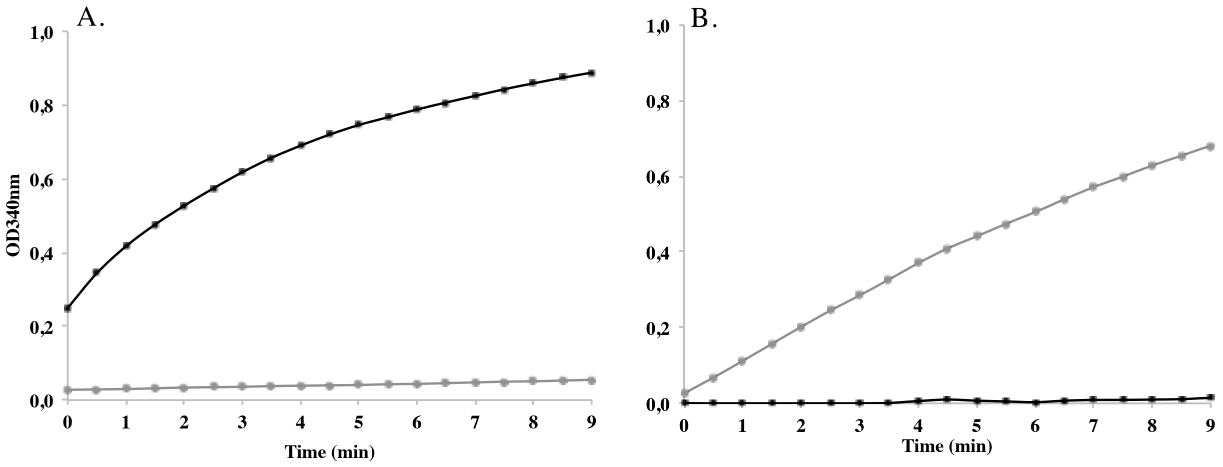

Figure S4: Biochemical parameters of *RgSPP*. The pH (A) and temperature (B) profiles for phosphorolysis of S6^{F}P by *RgSPP*.

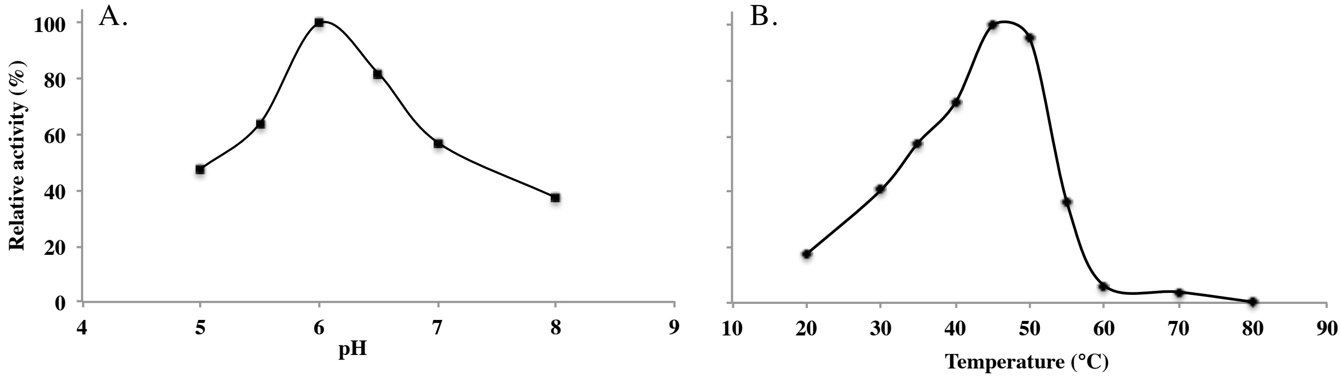

Figure S5: Sequence alignment of characterized GH13_18 members.

Multiple sequence alignment of characterized GH13_18 members using MAFFT EINSI advanced mode [47]. The secondary structures of AAO33821 from *B. adolescentis* have been solved using ESPrit [48]. Alpha helix is indicated with a loop while the beta strand is indicated with double horizontal bars. The fully, strongly and weakly conserved residues are marked with stars, colons and dots, respectively. The catalytic amino acid residues are in bold red. The transition state stabilizer from the catalytic triad is in bold green. The seven highly conserved domains (CSR) are squared in blue and the domain name is indicated. The position corresponding to the *Rg*SPP-Y355 residue is squared in orange.

```
1R7A:A_PDB
AAO33821_B.adol ------------------------------------------------------------
AAD40317_P.sacc ------------------------------------------------------------
BAF62433_B.long ------------------------------------------------------------
AAO84039_B.long ------------------------------------------------------------
AAN58596_S.muta M-----------------------------------------------------------
AAX33736_L.mese M-----------------------------------------------------------
BAA14344_L.mese M-----------------------------------------------------------
2207198A_L.mese M-----------------------------------------------------------
ABS59292_L.mese M-----------------------------------------------------------
AGK37834_L.reut M-----------------------------------------------------------
ADL69407_T.ther M-----------------------------------------------------------
ADP98617_M.adha M-----------------------------------------------------------
CAA61958_R.gnav ------------------------------------------------------------
ADH62582_M.silv MSSLTPELRQSILEHLGFLYG--------ERAPAVL----GRLEE-----ICSGFPAQRR
AEJ61152_S.ther M-----EPVDRMRELLSFIYGPETGRDTHEALHALLDGWRGRLPSPDEEYASGRLP----
AKR24578_E.coli M-------KQKITDYLDEIYG---GTFTATHLQKLV----TRLESAKR------LITQRR

                                                 CSR-VI
1R7A:A_PDB               ====                γγγγγγγγγ γγ      ====    ==
AAO33821_B.adol ----MKNKVQLITYADRLG---DG----TIKSMTDILR-TRFDGVYDGVHILP-FFTPFD
AAD40317_P.sacc ----MKNQVQLITYVDRLGGQRDGADLARLKKLLCEPG-QPLADVFGGVHLLP-FFHAID
BAF62433_B.long ----MKNKVQLITYADRLG---DG----TLSSMTDILR-TRFDGVYDGVHILP-FFTPFD
AAO84039_B.long ----MKNKVQLITYADRLG---DG----TLSSMADILR-TRFDGVYDGVHILP-FFTPFD
AAN58596_S.muta ---PIINKTMLITYADSLG----K----NLKELNENIE-NYFGDAVGGVHLLP-FFPS--
AAX33736_L.mese ---EIQNKAMFITYADSLG----K----NLKDVRKVLK-EDIGDAIGGVHLLP-FFPS--
BAA14344_L.mese ---EIQNKAMLITYADSLG----K----NLKDVHQVLK-EDIGDAIGGVHLLP-FFPS--
2207198A_L.mese ---EIQNKAMLITYADSLG----K----NLKDVHQVLK-EDIGDAIGGVHLLP-FFPS--
ABS59292_L.mese ---EIQNKAMLITYADSLG----S----NIKEVHQVLK-EDIGDAIGGVHLLP-FFPS--
AGK37834_L.reut ---PIKNEAMLITYSDSMG----K----NIKETHEVLK-NYIGDAIGGVHLLP-FFPS--
ADL69407_T.ther ---ALKNKVQLITYPDSLG----G----NLKTLNDVLE-KYFSDVFGGVHILP-PFPS--
ADP98617_M.adha ---LLKNAVQLICYPDRIG----N----NLKDLYTVVD-THLSEAIGGLHILP-FFPS--
CAA61958_R.gnav ----MNSKPMLNAYPDSLG----R----NLGDIVTLLKTEALQNVFSSFYILPSLYHS--
ADH62582_M.silv EGGWSEKDALLITYGDQIH----AEGEPPLQTLYDFLY-ERLRGVFSGVHLLP-FYPS--
AEJ61152_S.ther ---LDHTDAVLITYGDQFG----RKGEAPLATLGEFLR-EYLSGTMKGVHILP-FFPY--
AKR24578_E.coli KKHWDESDVVLITYADQFH----SNDLKPLPTFNQFYH-QWLQSIFSHVHLLP-FYPW--
                      .   :  * * :           :           :      .::**  :

                                       CSR-I
1R7A:A_PDB               =====      γγγγγγγγ   ======== ====  γγγγγγγγγγγγ
AAO33821_B.adol GADAGFDPIDHTKVDERLGSWDDVAELSK-THNIMVDAIVNHMSWESKQFQDVLAKGEES
AAD40317_P.sacc GADAGFDPIDHTLVDPRLGDWSDIKALTE-GLEVMGDVIVNHMSSESPQFQDFSAKGRES
BAF62433_B.long GADAGFDPIDHTKVDERLGSWDDVAELSK-THNIMVDAIVNHMSWESKQFQDVLEKGEES
AAO84039_B.long GADAGFDPIDHTKVDERLGSWDDVAELSK-THNIMVDAIVNHMSWESKQFQDVLEKGEES
AAN58596_S.muta TGDRGFAPIDYHEVDSAFGDWDDVKCLGE-KYYLMFDFMINHISRQSKYYKDYQEKHEAS
AAX33736_L.mese TGDRGFAPSDYTRVDSAFGDWSDVEALGE-EYYLMFDFMINHISRESVMYQDFKKNHDES
BAA14344_L.mese TGDRGFAPADYTRVDAAFGDWADVEALGE-EYYLMFDFMINHISRESVMYQDFKKNHDDS
2207198A_L.mese TGDRGFAPADYTRVDAAFGDWKDVEALGE-EYYLMFDFMINHISRESVMYQDFKKNHDDS
ABS59292_L.mese TGDRGFAPSDYTRVDATFGDWDDVEALGQ-EYYLMFDFMINHISRESEMYQDFKVNHDQS
AGK37834_L.reut TGDRGFAPYRYDVVDSAFGNWDDVEALGE-DYYLMFDFMINHISKKSEMYQDFKKKHDDS
ADL69407_T.ther SGDRGFAPITYSEIEPKFGTWYDIKKMAE-NFDILLDLMVNHVSRRSIYFQDFLKKGRKS
ADP98617_M.adha NADGGFSPLTHKEVDPKVGTWDDIEAFTA-KYDLCVDLTVNHISDESPEFTDFIANGFDS
CAA61958_R.gnav DLDRGFSVIDY-DLNEQLANREDLDRLKNMGIDLKLDFILNHASAQSPQFRDLVEKGEAS
ADH62582_M.silv TSDDGFSVVDFQRVDPELGTWTDIRIIAQ-DFRLMADLVCNHVSASSPWFQGFLQD--DP
AEJ61152_S.ther SSDDGFSVMDYRRVNPEWGTWDDVRRISE-DFRLMVDLVLNHCSAKSEWFRRFLQG--DP
AKR24578_E.coli SSDDGFSVIDYHQVASEAGEWQDIQQLGE-CSHLMFDFVCNHMSAKSEWFKNYLQQ--HP
                  * **    .  :    .   *:  :      :  *   ** *  *  :         .

1R7A:A_PDB        γγγ   γγγγ                γγγγγ          ======    ======
AAO33821_B.adol EYYPMFLTMSSVFP----------NGATEEDLAGIYRPRPGLPFTHYKFA--GKTRLVWV
AAD40317_P.sacc AYDGLFLTLDAVFP----------NGATERDLLTVYRPRPG-PAAELCDAEERRARILWT
BAF62433_B.long EYYPMFLTMSSVFP----------NGATEEDLAGIYRPRPGLPFTHYKFA--SKTRLVWV
AAO84039_B.long EYYPMFLTMSSVFP----------NGATEEDLAGIYRPRPGLPFTHYKFA--GKTRLVWV
AAN58596_S.muta AYKDLFLNWDKFWP------K---NRPTQEDVDLIYKRKDRAPKQEIQFAD-GSVEHLWN
AAX33736_L.mese KYKDFFIRWEKFWA------KAGENRPTQADVDLIYKRKDKAPTQEITFDD-GTTENLWN
BAA14344_L.mese KYKDFFIRWEKFWA------KAGENRPTQADVDLIYKRKDKAPTQEITFDD-GTTENLWN
2207198A_L.mese KYKDFFIRWEKFWA------KAGENRPTQADVDLIYKRKDKAPTQEITFDD-GTTENLWN
ABS59292_L.mese KYRDFFIRWEKFWQ------QAGPDRPTQADIDLIYKRRDKAPIQEITFAD-GMTEHLWN
AGK37834_L.reut KYNDFFIRWEKFWE------KAGKNRPTQEDVDLIYKRKDKAPKQEITFDD-GTTENLWN
ADL69407_T.ther EYADMFITLDKLWK------D---GKPVKGDIEKMFLRRTL-PYSTFKIEETGEEEKVWT
ADP98617_M.adha EYADLFVHVDKF------------GEISPDDMAKIHIRKEKEPFREVTLSD-GTKTRVWC
CAA61958_R.gnav AYRDFFIDWNHFWEGHGTMTEEGYIQPDESCLKQMFFRKPGLPILMVEFPD-GKKVPYWN
ADH62582_M.silv QYQGFFITVDP-----------------GTDLSTVFRPRALPLLTPFQTP--SGEKLVWT
AEJ61152_S.ther EYEDFFITVEP-----------------GTDLSGVFRPRALPLVHEFESA--KGPVLVWT
AKR24578_E.coli GFEDFFIAVDP-----------------QTDLSAVTRPRALPLLTPFQMRD-HSTRHLWT
                 :  :*:  .                     :  :   :                   *

                                  CSR-V                            CSR-II
1R7A:A_PDB                       ===     γγγγγγγγγγγγγγγγγ   ===== γγγ
AAO33821_B.adol SFTP----Q--------QVDIDTDSDKGWEYLMSIFDQMAASHVSYIRLDAVGYGAKEAG
AAD40317_P.sacc TFTA----A--------QIDIAVHHPQGRAYLESILQTFAANGIRMVRLDAVGYAIKKAG
BAF62433_B.long SFTP----Q--------QVDIDTDSAKGWEYLMSIFDQMAASHVRYIRLDAVGYGAKEAG
AAO84039_B.long SFTP----Q--------QVDIDTDSDKGWEYLMSIFDQMAASHVSYIRLDAVGYGAKEAG
AAN58596_S.muta TFGE----E--------QIDLDVTKEVTMDFIRSTIENLAANGCDLIRLDAFAYAVKKLD
AAX33736_L.mese TFGD----E--------QIDIDVNSAIAKEFIKTTLEDMVKHGANLIRLDAFAYAVKKVD
BAA14344_L.mese TFGE----E--------QIDIDVNSAIAKEFIKTTLEDMVKHGANLIRLDAFAYAVKKVD
2207198A_L.mese TFGE----E--------QIDIDVNSAIAKEFIKTTLEDMVKHGANLIRLDAFAYAVKKVD
ABS59292_L.mese TFGE----E--------QIDIDVNSQIAKAFIKATLEDMVQHGANLIRLDAFAYAVKKVG
AGK37834_L.reut TFGE----E--------QIDINVKSKVANEFFKETLIDMVKHGADMIRLDAFAYAIKKVG
ADL69407_T.ther TFGKTDPSE--------QIDLDVNSHLVREFLLEVFKTFSNFGVKIVRLDAVGYVIKKIG
ADP98617_M.adha TFTE----Q--------QIDLNYESDLAYQLMESYIGFLTSKGVNLLRLDAFGYTTKRIG
CAA61958_R.gnav TFYQ----EVHGRQYLGQMDVNIQSPKVWEFYRETLEKIASYGAAIVRLDAFAYAPKTPG
ADH62582_M.silv TFSP----D--------QTDLNYANPEVLLEVIEALLCYVRNGAGLIRLDAVGFIWKEIG
AEJ61152_S.ther TFSR----D--------QVDLNYANPRVLLEMIDIFLFYVSQGAQIIRLDAIAYLWKELG
AKR24578_E.coli TFSD----D--------QIDLNYRSPEVLLAMVDVLLCYLAKGAEYVRLDAVGFMWKEPG
                :*               * *:              :          :****..:  *  .
```

```
                                                     CSR-III
1R7A:A_PDB

AAO33821_B.adol TSCFMT-PKTFKLISRLREEGVKR--GLEILIEVHSYYKKQV--EI----ASKVDRVYDF
AAD40317_P.sacc ASCFMM-PETFGFIAEFAQAARAL--GIEVLVEIHAYYQRQI--EI----ASQVDWVYDF
BAF62433_B.long TSCFMT-PKTFKLISRLREEGVKR--GLEILIEVHSYYKKQV--EI----ASKVDRVYDF
AAO84039_B.long TSCFMT-PKTFKLISRLREEGVKR--GLEILIEVHSYYKKQV--EI----ASKVDRVYDF
AAN58596_S.muta TNDFFVEPEIWTLLDKVRDIAAVS--GAEILPEIHEHYTIQF--KI----ADHDYYVYDF
AAX33736_L.mese TNDFFVEPEIWDTLNEVREILTPL--KAEILPEIHEHYSIPK--KI----NDHGYFTYDF
BAA14344_L.mese TNDFFVEPEIWDTLNEVREILTPL--KAEILPEIHEHYSIPK--KI----NDHGYFTYDF
2207198A_L.mese TNDFFVEPEIWDTLNEVREILTPL--KAEILPEIHEHYSIPK--KI----NDHGYFTYDF
ABS59292_L.mese TNDFFVEPEIWAVLNEVRGILAPM--HAEILPEIHEHYTIPQ--KI----NAHGYFTYDF
AGK37834_L.reut TNDFFVEPEIWDLLNEVQDILAPY--KAIILPEIHEHYTIPQ--KI----SQHDFFIYDF
ADL69407_T.ther TSCFFVEPEIYEFLDWAKGQAASY--GIELLLEVHSQFEVQY--KL----AERGFLIYDF
ADP98617_M.adha TSCFLVEPEVYQILDWVNQVALKH--GAECLPEVHDHTSYQY--AI----SRRNMHPYGF
CAA61958_R.gnav KKNFLNDPETWELLQKIHALAEPL--GLTLLPEIHAATDEKIYETL----AEKGYATYDF
ADH62582_M.silv TSCMHL-EGAHRIVKLMRLVLDAVAPHVLLVSETNAPHRENI--SYFGNGHDEAQLVYQF
AEJ61152_S.ther TPCIHH-PKTHAVVKLFRAICEEVCPWVLIITETNVPHKENI--SYFGD-MDEAHLVYQF
AKR24578_E.coli TSCIHL-EKTHLIIKLLRSIIDNVAPGTVIITETNVPHKDNI--AYFGAGDDEAHMVYQF
                    :          :           : * :                 .    * *

                                                          CSR-IV
1R7A:A_PDB

AAO33821_B.adol ALPPLLLHALSTGHVEPVAHWTD-----IRPNNAVTVLDTHDGIGVI-DIGSDQLDRSLK
AAD40317_P.sacc ALPPLVLHAFEFKTAGALKRWIA-----VRPTNALTVLDTHDGIGII-DIGADASDRAAH
BAF62433_B.long ALPPLLLHSLFTGHVEPVAHWTE-----IRPNNAVTVLDTHDGIGVI-DIGSDQLDRSLK
AAO84039_B.long ALPPLLLHSLFTGHVEPVAHWTE-----IRPNNAVTVLDTHDGIGVI-DIGSDQLDRSLK
AAN58596_S.muta ALPMVTLYSLYSSKVDRLAKWLK-----MSPMKQFTTLDTHDGIGVV-DVK---------
AAX33736_L.mese ALPMTTLYTLYSGKTNQLAKWLK-----MSPMKQFTTLDTHDGIGVV-DAR---------
BAA14344_L.mese ALPMTTLYTLYSGKTNQLAKWLK-----MSPMKQFTTLDTHDGIGVV-DAR---------
2207198A_L.mese ALPMTTLYTLYSGKTNQLAKWLK-----MSPMKQFTTLDTHDGIGVV-DAR---------
ABS59292_L.mese ALPMTVLYTLYSGKTNRLANWLK-----QSPMKQFTTLDTHDGIGVV-DAR---------
AGK37834_L.reut TLPMTTLYTLYSGKTNRLAKWLK-----MSPMKQFTTLDTHDGIGVV-DAK---------
ADL69407_T.ther ILPFTVLYTLINKSNEMLYHYLK-----NRPINQFTMLDCHDGIPVKPDLD---------
ADP98617_M.adha ALPPLLLYSLLDANSTYLKNWLR-----MCPRNMVTVLDTHDGICIP-DVE---------
CAA61958_R.gnav FLPGLVIDAIENRRGTYLAAWAKEIV--EKKISTVNMLGCHDGIPLL-DLK---------
ADH62582_M.silv PLPPLVMHTFRTGDASKLAGWAAGLTLPSERTTFFNFLASHDGIGVV-PAG---------
AEJ61152_S.ther ALPPLVLDAFLRKDVSYLREWARTIDTYGGKVSYFNFLASHDGIGVL-PAR---------
AKR24578_E.coli SLPPLVLHAVQKQNVEALCAWAQNLTLPSSNTTWFNFLASHDGIGLN-PLR---------
                  **   : :.        :   :           .  ..  *   ****  :

1R7A:A_PDB

AAO33821_B.adol -GLVPDEDVDNLVNTIHANTHGESQAATG--AAASN-LDLYQVNSTYYSALGCND-----
AAD40317_P.sacc PGLVPPEELDALVERIHAASQGQSRKPTG--AAASN-LDLYQVNCSFFDAMGRNE-----
BAF62433_B.long -GLVPDEDVDNLVNTIHANTHGESQAATG--AAASN-LDLYQVDSTYYSALGCND-----
AAO84039_B.long -GLVPDEDVDNLVNTIHANTHGESQAATG--AAASN-LDLYQVNSTYYSALGCND-----
AAN58596_S.muta -DILTDEEITYTSNELYKVGANVNRKYST--AEYNN-LDIYQINSTYYSALGDDD-----
AAX33736_L.mese -DVLTDEEIDYASEELYKVGANVKKTYSS--ASYND-LDIYQINSTYYSALGNDD-----
BAA14344_L.mese -DILTDDEIDYASNELYKVGANVKKTYSS--ASYNN-LDIYQINSTYYSALGNDD-----
2207198A_L.mese -DILTDDEIDYASEQLYKVGANVKKTYSS--ASYNN-LDIYQINSTYYSALGNDD-----
ABS59292_L.mese -DILTDEEIDYASEELYKVGANVKKTYSS--AAYNN-LDIYQINSTYYSALGNDD-----
AGK37834_L.reut -DILTDDEIEYASNELYKVGANVKRKYSS--AEYNN-LDIYQINSTYYSALGDDD-----
ADL69407_T.ther -GLIDTKKAKEVVDICVQRGANLSLIYGDKYKSEDG-FDVHQINCTYYSALNCDD-----
ADP98617_M.adha -GVLPDEKIKVLIDNIDARSADPIMRRSA--ANIHSVGAIYQLTCTFYDALMQND-----
CAA61958_R.gnav -GLLPEEEIQSLIERIVSRGGMVKNLHGQ-----KN--LYYQVNATYYSALGASD-----
ADH62582_M.silv -GILQPEEIAALVRQALEHGGRVNHKDTP-----DG-PVPYELCLTLFDALSNPNSDEAE
AEJ61152_S.ther -GILPDEYIDAMIEAVKDRGGLISYKSTP-----QG-EVPYELNINYLSAISESHLDRPT
AKR24578_E.coli -GLLPESEILELVEALQQEGALVNWKNNP-----DGTRSPYEINVTYMDALSRRESSDEE
                  .::  .                                .     :::   .  .*:    .

                                       CSR-VII
1R7A:A_PDB

AAO33821_B.adol ----QHYIAARAVQFFLPGVPQVYYVGALAGKNDMELLRKTNNG--RDINRHYYSTAEID
AAD40317_P.sacc ----TAYLLARAIQLFLPGVPQVYYVGLLAGHNDMELLARTQVG--RDINRHYYDAAEIA
BAF62433_B.long ----QHYLAARAVQFFLPGVPQVYYVGALAGRNDMELLRRTNNG--RDINRHYYSTAEID
AAO84039_B.long ----QHYLAARAVQFFLPGVPQVYYVGALAGRNDMELLRRTNNG--RDINRHYYSTAEID
AAN58596_S.muta ----QKYFLARLIQAFAPGIPQVYYVGFLAGKNDLELLESTKEG--RNINRHYYSSEEIA
AAX33736_L.mese ----AAYLLSRIFQVFAPGIPQIYYVGLLAGESDIELLESSKEG--RNINRHYYSVDEVK
BAA14344_L.mese ----AAYLLSRVFQVFAPGIPQIYYVGLLAGENDIALLESTKEG--RNINRHYYTREEVK
2207198A_L.mese ----AAYLLSRVFQVFAPGIPQIYYVGLLAGENDIALLESTKEG--RNINRHYYTREEVK
ABS59292_L.mese ----AAYLLSRVFQVFAPGIPQIYYVGLLAGENDIDLLESSKEG--RNINRHYYTREEIK
AGK37834_L.reut ----KAYLLSRAFQVFAPGIPMVYYVGLLAGSNDLELLEKTKEG--RNINRHYYTKEEVA
ADL69407_T.ther ----DAYLAARAIQFFTPGIPQVYYVGLLAGVNDFEAVKKTKEG--REINRHNYGLKEIE
ADP98617_M.adha ----DAYIAARAIQFFTPGIPQVYYVGLLAGCNDHELMEQSGEL--RDINRHYYTLEEVE
CAA61958_R.gnav ----EKMLLARAIQMFMPGKPQVWYLDLFAGKNDHEAVRRAGESGHKEINRTNLSTDQIT
ADH62582_M.silv DLKIARFLAANVILLSLQGIPGVYIHSLFGSPSDHAGFEESGIP--RRLNRHKFTKAELE
AEJ61152_S.ther --RARKFLASQAVMLSLVGMPGIYVHSLLGSENWREGVEKTGMN--RTINRQKLSYEGVL
AKR24578_E.coli --RCARFILAHAILLSFPGVPAIYIQSILGSRNDYAGVEKLGYN--RAINRKKYHSKEIT
                         :  :.  .      *  *  ::   .  :. .      .        :  :**        :

1R7A:A_PDB

AAO33821_B.adol ENLKRP------VVKALNALAKFRNELDAF--DGTFSYTTDDDTSISFTWRGE--TSQAT
AAD40317_P.sacc SDCERP------VVRRLIELIRLRNRHPAF--GGVFSVEAGGDDELHLRWQQGG--DWGP
BAF62433_B.long ENLERP------VVKALNALAKFRNELPAF--DGEFSYEVDGDTSITFRWTAADGTSTAA
AAO84039_B.long ENLERP------VVKALNALAKFRNELPAF--DGEFSYEVDGDTSITFRWTAADGTSTAA
AAN58596_S.muta KEVKRP------VVKALLNLFTYRNQSAAFDLDGRIEVETPNEATIVIERQNKDGSHIAK
AAX33736_L.mese EEVKRP------VVAKLLKLLSWRNNFAAFDLDGSIDVETPSDTTIKITRKDKSGENVAV
BAA14344_L.mese SEVKRP------VVANLLKLLSWRNESPAFDLAGSITVDTPTDTTIVVTRQDENGQNKAV
2207198A_L.mese SEVKRP------VVANLLKLLSWRNESPAFDLAGSITVDTPTDTTIVVTRQDENGQNKAV
ABS59292_L.mese SAVKRP------VVADLLALLSWRNQFSAFALDGTITVETPSEHDIKITRTDHSGDNIAI
AGK37834_L.reut QEVQRP------VVKNLLDLLAWRNKFAAFDLDGSIDVETPTETTIKITRKDKDGKNVAV
ADL69407_T.ther ESVQKN------VVQRLLKLIRFRNEYEAF--NGEFFIEDCRKDEIRLTWKKDD--KRCS
ADP98617_M.adha QDIQKP------VVQRLLSLMKFRSNYPAF--DGHFELNYSNNSSVAMAWRHGD--YYCH
CAA61958_R.gnav EGLKKD------VVQKQLALIRMRNTHKAFSEGAEVAI-SGGERSLEIRWEYNS--AFAT
ADH62582_M.silv ERLADPASRAAKILAAYSHLLRVRSMHPAFHPNAPQRI-LPSTEVLRIVRGEGD--QAVG
AEJ61152_S.ther EELRDPESLRSMVFEGYLDMLAARRKSRAFDPRGMQEVLEAPETVFALLRRSPDATEEVL
AKR24578_E.coli RELNDEATLRHAVYHELSRLITLRRSHNEFHPDNNFTIDTINSSVMRIPRSNADG-NCLT
                                 :        :    *       *               . .
```

```
1R7A:A_PDB      === ϒϒϒ          =======  =====
AAO33821_B.adol LTFEPKRGLGVDNTTPVAMLEWEDSAGDHRSDDLIA------------------------
AAD40317_P.sacc LRVNFA-----SLDH---ELSCSRRRRD-RAFAFS-------------------------
BAF62433_B.long LTFEPGRGLGTDNATPVASLAWSDAAGDHETRDLLA------------------------
AAO84039_B.long LTFEPGRGLGTDNATPVASLAWSDAAGDHETRDLLA------------------------
AAN58596_S.muta AEINLQ-----DMTY---RVTENDQTIS-FE-----------------------------
AAX33736_L.mese LVANAA-----DKTF---TITANGEEIL-ANTEADK------------------------
BAA14344_L.mese LTADAA-----NKTF---EIVENGQTVM-SSDNLTQ------------------------
2207198A_L.mese LTADAA-----NKTF---EIVENDQTVM-SSDNLTQ------------------------
ABS59292_L.mese LLANAK-----TRTF---VITAMGKTVL-QNK----------------------------
AGK37834_L.reut LDADAA-----NKTF---TITANGEKVM-EQK----------------------------
ADL69407_T.ther LFIDLK-----TYKT---TIDYINENGE-EVKYLV-------------------------
ADP98617_M.adha LFVDLN-----FKTV---KVTYTDVETG-ETRHLEC------------------------
CAA61958_R.gnav LYVNFE-----SGTY---TIVESK------------------------------------
ADH62582_M.silv CYINVT-----DRPQ---VVSRIGKNLI-TGQWFTG-------------------VLKPY
AEJ61152_S.ther CLINVS-----HIEQECVFPSSIFRTAP-DAHLFTELTSGDTLVPYREDEDRFSISLGGY
AKR24578_E.coli GLFNVS-----KNIQHVNITNLHGRDLI-SEVDILG----------------NEITLRPW
                   :

1R7A:A_PDB
AAO33821_B.adol ----NPPVVA--
AAD40317_P.sacc ------------
BAF62433_B.long ----NPPIADID
AAO84039_B.long ----NPPIADID
AAN58596_S.muta ------------
AAX33736_L.mese ----QQL-----
BAA14344_L.mese ----N-------
2207198A_L.mese ----N-------
ABS59292_L.mese ------------
AGK37834_L.reut ------------
ADL69407_T.ther ------------
ADP98617_M.adha ------------
CAA61958_R.gnav ------------
ADH62582_M.silv QAAWIID-----
AEJ61152_S.ther EVLWLTPYRDKG
AKR24578_E.coli QVMWIK------
```
